# Chondrocyte‐Targeted Nanoparticles Loaded with *N*‐Acetylcysteine Protect Articular Cartilage and Attenuate Osteoarthritis by Inhibiting Ferroptosis via Glutathione Maintenance

**DOI:** 10.1002/smsc.202500440

**Published:** 2025-11-28

**Authors:** Shaoyi Wang, Fujian Zhang, Xiaocong Zhou, Jie Yang, Zhe Li, Songlin Li, Qunshan Lu, Houyi Sun, Peilai Liu

**Affiliations:** ^1^ Department of Orthopaedic Surgery Qilu Hospital Cheeloo College of Medicine Shandong University 107 Wenhuaxi Road Jinan Shandong 250012 P. R. China; ^2^ Qilu Hospital of Shandong University Spine and Spinal Cord Disease Research Center‐ ICMRS Collaborating Center for Orthopaedic translational Research Shandong University Jinan Shandong 250012 P. R. China; ^3^ Health Management Centre The First Affiliated Hospital of Shandong First Medical University Jinan Shandong 250012 P. R. China

**Keywords:** ferroptosis, glutathione, *N* ‐acetylcysteine, nanoparticle, osteoarthritis

## Abstract

Osteoarthritis (OA) is a degenerative joint disease characterized by cartilage degradation. Abnormal mechanical loading exacerbates intracellular ROS accumulation and glutathione (GSH) depletion. While *N*‐acetylcysteine (NAC) has potent antioxidant properties, its therapeutic potential in OA is limited by rapid degradation and poor intraarticular retention. In this study, chondrocyte‐targeted, chondroitin sulfate (CS)‐modified PLGA nanoparticles (CS‐NAC‐NPs) is developed for sustained and localized delivery of NAC. These nanoparticles exhibit excellent physical and chemical properties, biocompatibility, and chondrocyte targeting capabilities. In vitro, CS‐NAC‐NPs attenuated mechanical stress‐induced ROS accumulation, preserved mitochondrial integrity, restored GSH levels, and suppressed ferroptosis, as evidenced by increased GPX4 expression and improved chondrocyte viability. In a murine model of OA, intraarticular injection of CS‐NAC‐NPs significantly reduced cartilage degradation and osteophyte formation, improved histological scores, and maintained extracellular matrix homeostasis more effectively than free NAC or nontargeted NAC‐NPs. Notably, the therapeutic effect is abolished in GPX4‐deficient mice, confirming that CS‐NAC‐NPs act via GPX4‐mediated ferroptosis inhibition. Furthermore, in vivo tracking demonstrated excellent joint retention and no off‐target toxicity, underscoring their translational safety. This study introduces a novel nanotherapeutic platform that couples biomechanical targeting with redox‐responsive delivery to modulate ferroptosis, offering a promising disease‐modifying approach for OA treatment.

## Graphical abstract

Chondrocyte‐targeted CS‐NAC nanoparticles restore redox balance, inhibit ferroptosis, and protect cartilage under mechanical stress. This nanoplatform enhances joint retention, elevates GPX4 activity, and attenuates osteoarthritis progression, offering a promising disease‐modifying therapeutic strategy.

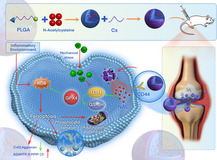

## 1 Introduction

Osteoarthritis (OA) represents a profound and escalating global health burden, affecting over 500 million individuals worldwide and ranking as a leading cause of chronic pain and functional disability.^[^ ^1^, ^2^ ^]^ This degenerative joint disease is characterized by the progressive erosion of the joint cartilage. The main manifestations include: a decrease in the activity and death of chondrocytes, as well as the presence of type II collagen and aggrecan.^[^ ^1^, ^3^, ^4^, ^5^ ^]^ While ageing and inflammation are established risk factors, abnormal mechanical stress is increasingly recognized as a critical instigator of OA pathogenesis.^[^ ^6^, ^7^, ^8^ ^]^ Excessive joint loading from trauma, obesity, or malalignment initiates a destructive cascade wherein biomechanical signals are transduced into biochemical dysfunction. Crucially, studies confirm that abnormal mechanical stress activates the mechanosensitive ion channel Piezo1 on chondrocytes, triggering pathological calcium (Ca^2^^+^) influx that disrupts mitochondrial integrity and amplifies reactive oxygen species (ROS) generation.^[^ ^6^ ^]^ This mechanotransduction pathway directly impairs glutathione (GSH) biosynthesis and recycling, depleting the primary intracellular antioxidant reservoir. The resulting oxidative stress overwhelms chondrocyte defenses, propagating a vicious cycle of extracellular matrix (ECM) degradation, proinflammatory mediator release, and eventual cell death.^[^ ^9^, ^10^ ^]^

*N*‐acetylcysteine (NAC), a cysteine precursor and widely used antioxidant, has demonstrated potent ROS‐scavenging capacity through both direct free radical neutralization and enhancement of intracellular glutathione (GSH) biosynthesis.^[^ ^11^, ^12^ ^]^ NAC has shown protective effects in oxidative stress‐related diseases by attenuating ROS‐induced injury, promoting matrix synthesis, and inhibiting inflammatory cascades.^[^ ^12^, ^13^ ^]^ However, the clinical translation of NAC remains limited due to its physicochemical instability in biological environments. Intraarticularly administered NAC undergoes rapid hydrolysis and clearance, resulting in a short half‐life and limited therapeutic retention. Therefore, a delivery strategy capable of stabilizing NAC, enabling sustained release, and improving local bioavailability is essential to fully realize its therapeutic potential in OA.

Biodegradable polymeric nanoparticles, such as those made from poly (lactic‐co‐glycolic acid) (PLGA), provide an effective platform for protecting labile drugs like NAC and enabling controlled drug release.^[^ ^14^, ^15^ ^]^ Nonetheless, conventional PLGA nanoparticles lack specific affinity for cartilage tissue and are subject to rapid clearance from the joint cavity. To enhance cartilage targeting and improve chondrocyte uptake, surface modification of nanoparticles with targeting ligands is a rational strategy. Chondroitin sulfate (CS), a sulfated glycosaminoglycan naturally present in cartilage, is an attractive candidate due to its ability to bind CD44 receptors, which are upregulated on stressed or inflamed chondrocytes.^[^ ^16^, ^17^, ^18^ ^]^ Additionally, CS interacts electrostatically with cartilage matrix components such as type II collagen, particularly in mechanically compromised cartilage, thereby promoting matrix retention and cellular internalization.

In this study, we developed CS‐modified NAC‐loaded PLGA nanoparticles (CS‐NAC‐NPs) to achieve joint‐targeted, redox‐responsive therapy for OA. We hypothesized that CS‐NAC‐NPs would improve NAC stability and intraarticular retention, enhance chondrocyte uptake through CD44‐ and ECM‐mediated targeting, and suppress mechanical stress‐induced ROS accumulation by restoring GSH levels. Through in vitro and in vivo experiments, we evaluated the physicochemical characteristics, cartilage‐targeting efficiency, therapeutic efficacy, and underlying mechanism of CS‐NAC‐NPs. Our results provide a novel strategy for the treatment of OA through ferroptosis inhibition and targeted antioxidant delivery.

## 2 Results

### 2.1 Preparation and Characterization of Nanoparticles

**Figure 1:**
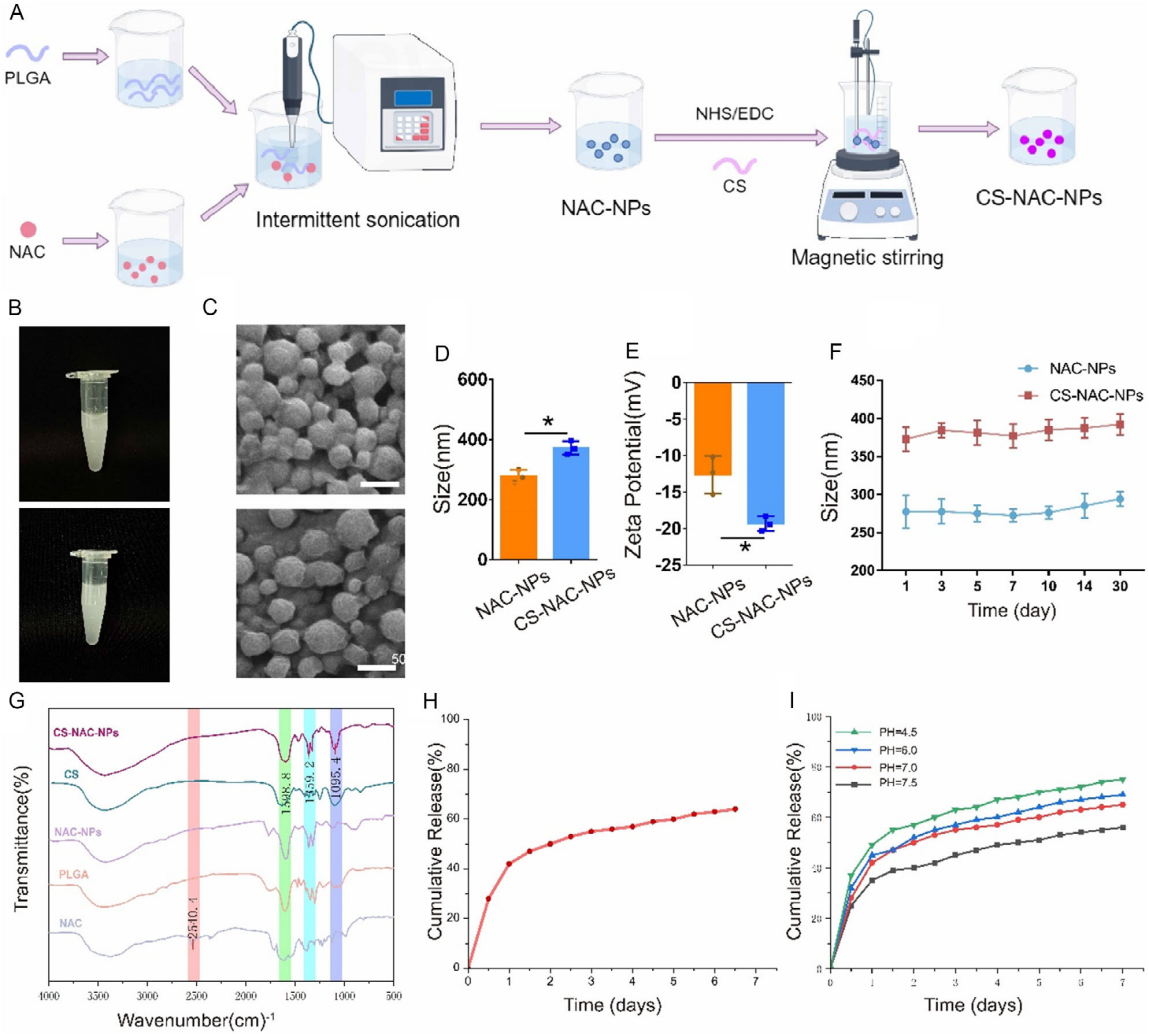
Preparation and characterization of NAC‐loaded nanoparticles. A) Schematic illustration of the preparation process for NAC‐NPs and CS‐NAC‐NPs B) Photographs of NAC‐NPs and CS‐NAC‐NPs suspensions in water showing uniform milky‐white appearance. C) SEM images showing irregular spherical morphology of both types of nanoparticles. D,E) Size distribution and zeta potential analysis of NAC‐NPs and CS‐NAC‐NPs. F) Colloidal stability of NAC‐NPs and CS‐NAC‐NPs was measured by DLS. G) FTIR spectra of NAC, NAC‐NPs, CS, and CS‐NAC‐NPs confirming drug encapsulation and surface modification. H) In vitro release profile of NAC from CS‐NAC‐NPs under different pH conditions, demonstrating sustained and pH‐sensitive drug release over 7 days. I) pH‐dependent release kinetics showing accelerated NAC release under acidic conditions (pH 4.5), indicative of pH‐sensitive release behavior. Data represent mean ± SD; ^*^ *P* < 0.05, ^**^ *P* < 0.01.

We prepared NAC‐NPs by employing a double emulsion (W/O/W) solvent evaporation method using PLGA as the carrier material (**Figure** **1**A). As summarized in Table S1, Supporting Information, NPs had optimum particle size, zeta potential, drug loading and encapsulation efficiency in the present of NAC (30 mg mL^−1^) and PLGA (50 mg mL^−1^). Thus, this combination was selected for further experiments. Subsequently, CS was grafted onto the surface of the PLGA nanoparticles via amide bond formation between the amino groups of CS and the carboxyl groups of PLGA, using EDC/NHS as coupling agents. This process yielded CS‐NAC‐NPs. Both types of nanoparticles formed uniform, milky‐white suspensions when dissolved in water (Figure 1B). Scanning electron microscopy (SEM) images revealed that both nanoparticle types were irregularly spherical in morphology (Figure 1C). As shown in Figure 1D,E, NAC‐NPs exhibited an average diameter of 277 nm with a surface charge of –12.6 mV, while CS‐NAC‐NPs displayed an increased diameter of 372.3 nm and a surface charge of –19.33 mV (Table S2, Supporting Information). Both nanoparticle formulations demonstrated remarkable stability and uniform dispersion in aqueous solution (Figure 1F). Fourier‐transform infrared (FTIR) spectroscopy was used to characterize the functional groups present at different vibrational frequencies (Figure 1G). In the NAC spectrum, a distinctive peak at 2540 cm^−^^1^, attributed to –SH stretching, was observed. A peak at 1598 cm^−^^1^ corresponding to –C=O stretching confirmed the presence of PLGA. The disappearance of the –SH peak in the NAC‐NP spectrum indicated strong interactions between the thiol group of NAC and the nanoparticle matrix, confirming successful drug encapsulation. Compared to NAC‐NPs, both CS and CS‐NAC‐NPs exhibited a prominent peak at 1100 cm^−^^1^, corresponding to C–O stretching vibrations. Additionally, the relatively flattened shape in the 3200–3500 cm^−^^1^ region of the CS‐NAC‐NPs spectrum further supported the successful surface modification of the nanoparticles with chitosan. Next, we evaluated the release efficiency of CS‐NAC‐NPs. As shown in Figure 1H, ≈60% of the encapsulated NAC was released over a continuous 7‐day incubation period. Notably, the drug release rate significantly increased under acidic conditions. In particular, a faster release profile was observed in acidic buffer (pH 4.5), indicating pH‐sensitive release behavior of the nanoparticles (Figure 1I).

### 2.2 Biocompatibility and Chondrocyte‐Targeting Properties of the Nanoparticles

**Figure 2:**
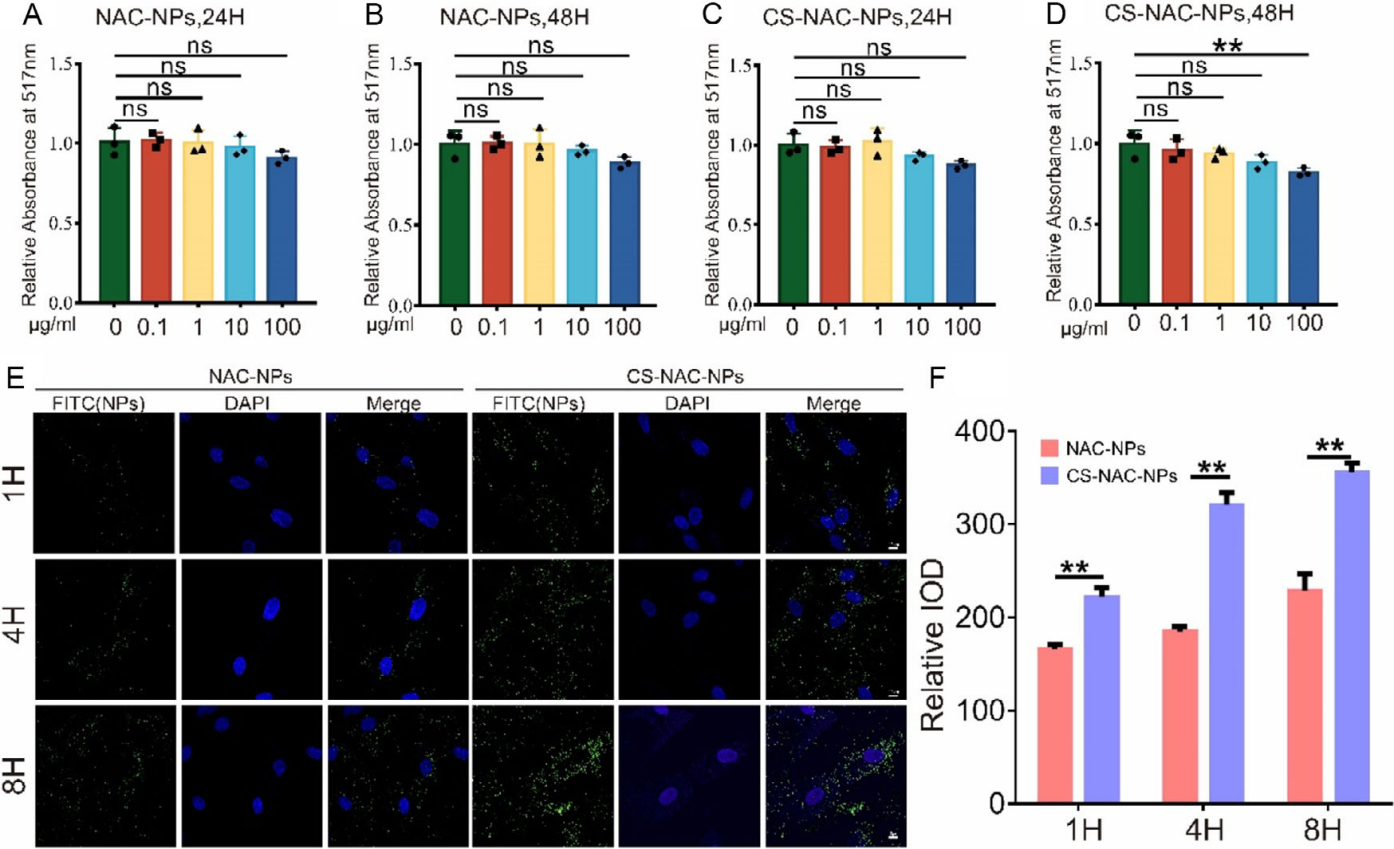
Biocompatibility and chondrocyte‐targeting properties of NAC‐NPs and CS‐NAC‐NPs. A,B) Cell viability of chondrocytes after incubation with varying concentrations of NAC‐NPs for 24 h (A) and 48 h (B), assessed using a Calcein‐AM/PI Viability/Cytotoxicity Assay Kit. C,D) Cell viability of chondrocytes treated with different concentrations of CS‐NAC‐NPs for 24 h (C) and 48 h (D). E,F) Quantitative analysis of intracellular fluorescence intensity to assess nanoparticle uptake by chondrocytes. CS‐NAC‐NPs demonstrated significantly enhanced cellular internalization at all time points compared to NAC‐NPs, indicating improved chondrocyte‐targeting ability. Scale bar = 10 μm. Data represent mean ± SD; ^*^ *P* < 0.05, ^**^ *P* < 0.01. ns: not significant.

We next evaluated the biocompatibility of the two nanoparticle formulations. Chondrocytes were incubated with various concentrations of NAC‐NPs and CS‐NAC‐NPs for 24 and 48 h, respectively. Cell viability was assessed to determine cytotoxicity. As shown in **Figure** **2**A–D, both nanoparticle types exhibited favorable biocompatibility at most tested concentrations, with the exception of CS‐NAC‐NPs at 100 μg mL^−1^, which showed a slight decrease in cell viability. To assess cellular uptake, we measured the intracellular fluorescence intensity of the nanoparticles. Across all time points, the fluorescence intensity in chondrocytes treated with CS‐NAC‐NPs was consistently higher than that observed in the NAC‐NPs group, indicating enhanced cellular internalization of CS‐modified nanoparticles (Figure 2E,F). To verify whether the CS‐NAC‐NPs enhanced chondrocyte targeting through CD44, small interfering RNA (siRNA) was used to knock down CD44 expression in chondrocytes. IF and WB analysis confirmed an efficient reduction of CD44 expression (Figure S1A–D, Supporting Information). Microscopic observation revealed that after CD44 knockdown, there was no significant difference in intracellular nanoparticle fluorescence between CS‐NAC‐NPs and NAC‐NPs at either 1 h or 8 h (Figure S1E,F, Supporting Information). The results demonstrated that CS modification enhanced the chondrocyte‐targeting capability of the nanoparticles through CD44‐mediated interactions. In vivo experiments, we examined the fluorescence intensity of chondrocytes in knee joint tissue sections at 24 and 48 h after the injection of the two types of nanoparticles. As shown in Figure S2A,B, Supporting Information, chondrocytes in the CS‐NAC‐NPs–injected group exhibited markedly stronger fluorescence signals compared with those in the NAC‐NPs group. These findings indicate that chitosan modification facilitates the cellular uptake of nanoparticles by chondrocytes in vivo.

**Figure 3:**
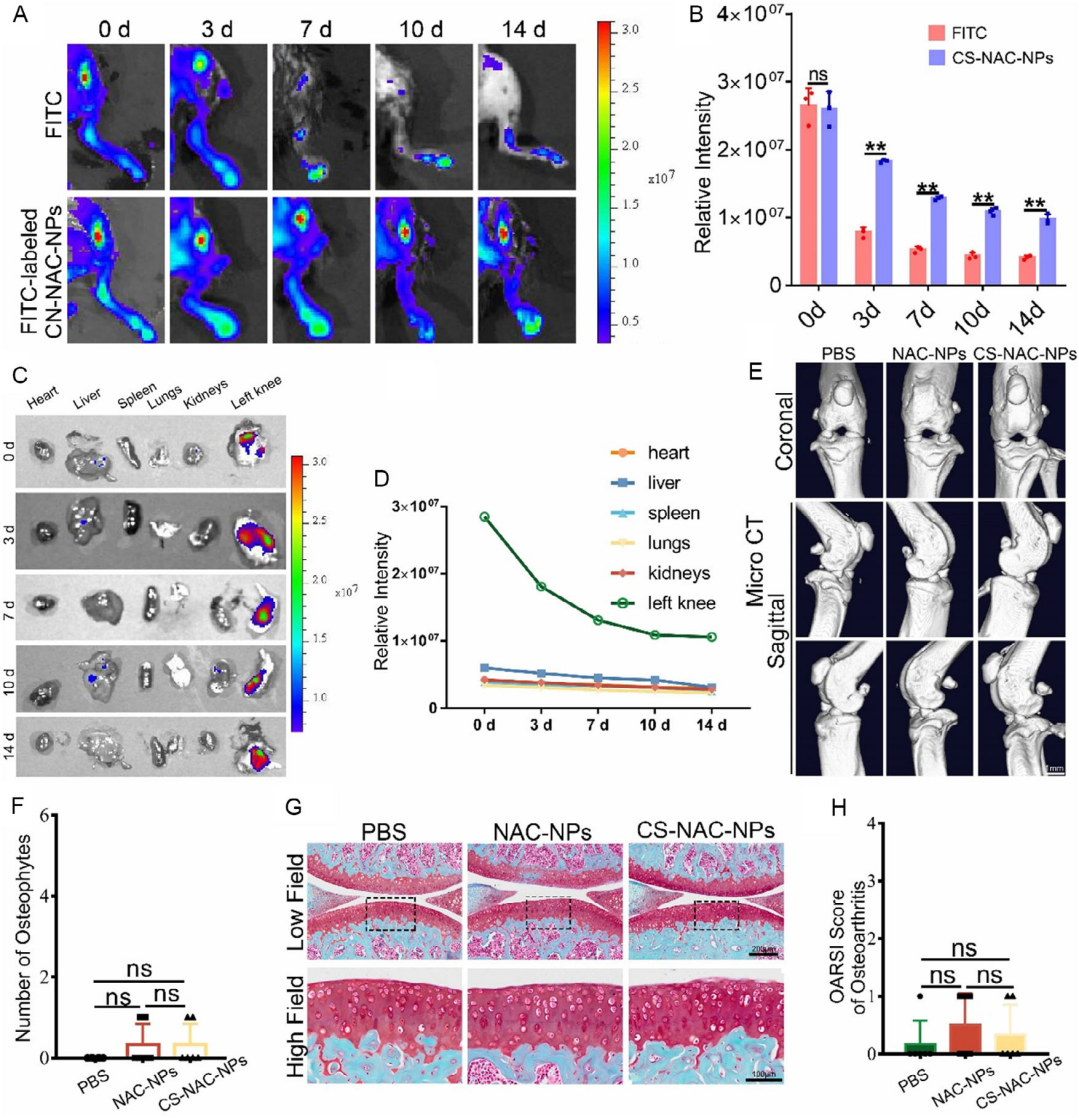
In vivo retention and biosafety evaluation of nanoparticles. A) Representative IVIS images showing the fluorescence signals in mouse knee joints after intraarticular injection of free FITC or FITC‐labeled CS‐NAC‐NPs at different time points (0, 1, 3, 7, and 14 days). B) Quantification of fluorescence intensity in the knee joints (*n* = 3 per group). C) Ex vivo fluorescence images of major organs (heart, liver, spleen, lung, kidney) and knee joint tissues collected at various time points postinjection of FITC‐labeled nanoparticles. D) Quantification of fluorescence intensity in major organs (heart, liver, spleen, lung, kidney) and knee joint (*n* = 3 per group). E) Representative Micro‐CT images of knee joints 30 days after intraarticular injection of PBS, NAC‐NPs, or CS‐NAC‐NPs. Scale bar = 1 mm. F) Quantification of osteophyte formation in each group. G) Safranin O staining of cartilage sections showed no observable cartilage degradation or matrix loss across all groups. H) OARSI scores indicated no statistically significant cartilage damage following nanoparticle treatment, confirming intraarticular biocompatibility (*n* = 5). All quantitative data are presented as mean ± SD. ^*^ *P* < 0.05, ^**^ *P* < 0.01. ns: not significant.

We further evaluated the in vivo retention of the nanoparticles. FITC‐labeled nanoparticles and free FITC were intraarticularly injected into the left knee joints of mice, and fluorescence signals were monitored at various time points using an in vivo imaging system (IVIS). As shown in **Figure** **3**A,B, the fluorescence signal in the free FITC group rapidly diminished and was nearly undetectable by day 7. In contrast, the knees of mice injected with FITC‐labeled nanoparticles retained a visible fluorescence signal that persisted with relatively stable intensity up to day 14, demonstrating the superior joint retention capability of the nanoparticles. Additionally, at each time point, three mice from each group were randomly selected and sacrificed. Major organs (heart, liver, spleen, lung, and kidney) and the injected knee joints were harvested for fluorescence analysis. As shown in Figure 3C,D, no detectable fluorescence was observed in any major organs at any time point, indicating minimal systemic distribution of the nanoparticles. To further assess the in vivo biosafety of the nanoparticles, knee joint tissues were collected 30 days after intraarticular injection. As shown in Figure 3E,F, compared to untreated controls, neither NAC‐NPs nor CS‐NAC‐NPs induced significant osteophyte formation. Safranin O staining of the joint sections (Figure 3G,H) revealed no apparent cartilage damage in nanoparticle‐injected joints relative to controls. Similarly, heart, liver, spleen, lung, and kidney tissues were harvested and sectioned for histological analysis 30 days postinjection to evaluate the systemic biosafety of the nanoparticles. As shown in Figure S3, Supporting Information, no notable histopathological abnormalities were observed in either the nanoparticle‐treated groups or the control group. Specifically, cardiac tissue exhibited well‐aligned myocardial fibers with intact cellular morphology; hepatic architecture was preserved with normal hepatocyte arrangement and no evidence of necrosis or inflammation; splenic tissue showed normal white and red pulp distribution; pulmonary tissue displayed clear alveolar structures without signs of infiltration or edema; and renal tissue demonstrated intact glomeruli and renal tubules without pathological alterations, indicating good systemic biocompatibility of the nanoparticles. The immunohistochemical staining analysis showed that there was no significant difference in the levels of inflammatory factor IL‐1β (Figure S4, Supporting Information) and TNF‐α (Figure S5, Supporting Information) in the heart, liver, spleen, lung and kidney among all groups.

### 2.3 Protective Effects of NAC‐NPs and CS‐NAC‐NPs on Chondrocytes

**Figure 4:**
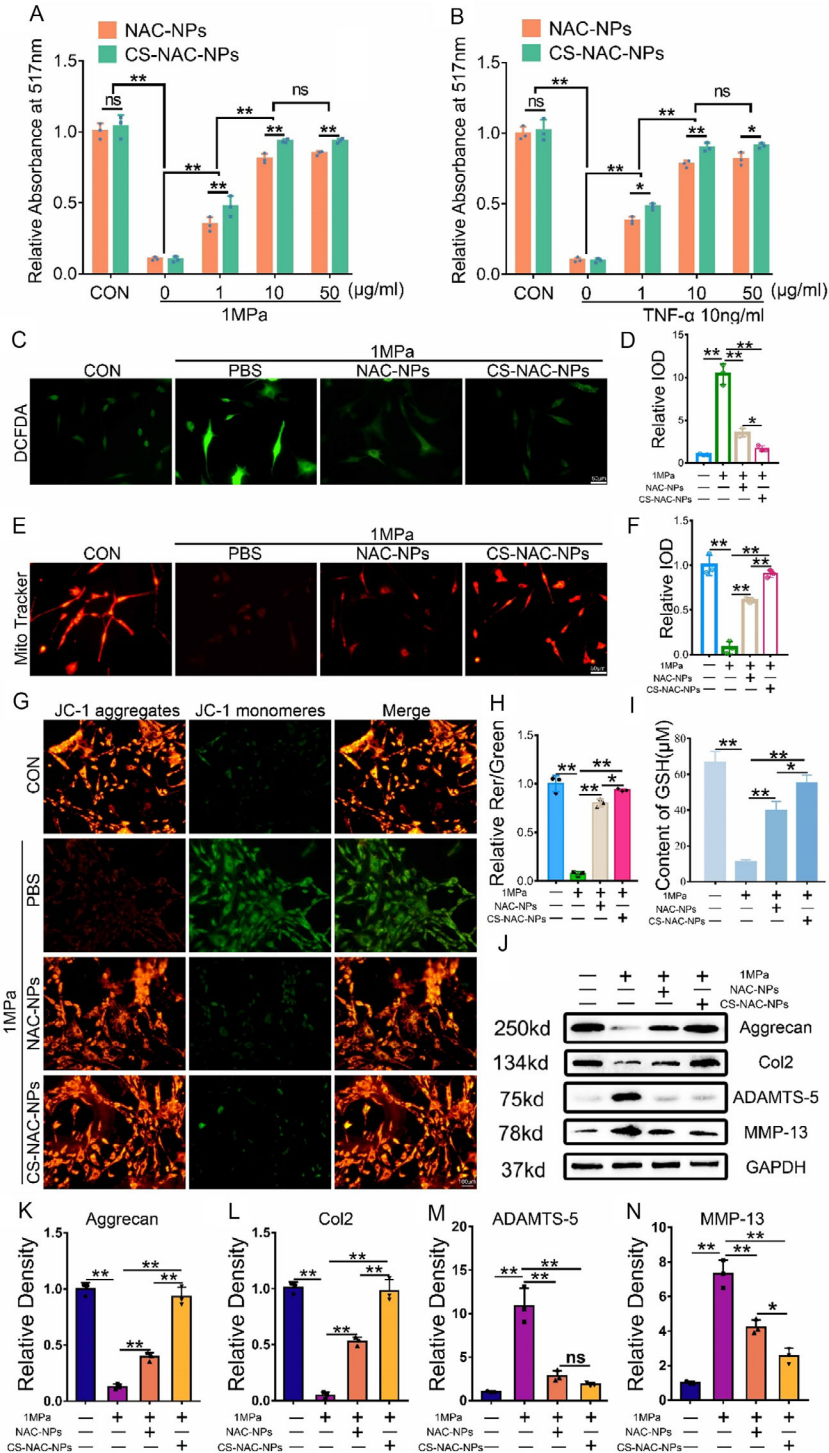
Protective effects of NAC‐NPs and CS‐NAC‐NPs on chondrocytes. A,B) Cell viability of chondrocytes treated with NAC‐NPs or CS‐NAC‐NPs (10 μg mL^−1^) for 24 or 48 h under 1 MPa mechanical stress and TNF‐α stimulation (10 ng mL^−1^), assessed by a Calcein‐AM/PI Viability/Cytotoxicity Assay Kit. C) Representative images of ROS levels in chondrocytes. Scale bar = 50 μm. D) Quantitative analysis of fluorescence intensity (*n* = 3 for each group). E) Representative fluorescence images of mitochondria in chondrocytes. Scale bar = 50 μm. F) Quantitative analysis of fluorescence intensity (*n* = 3 for each group). G) Mitochondrial membrane potential was detected by JC‐1 assay. Scale bar = 50 μm. H) The relative IOD ratio of red fluorescence to green fluorescence was used for quantitative analysis (*n* = 3 for each group). I) Intracellular glutathione (GSH) levels detected by a GSH assay kit. CS‐NAC‐NPs more effectively restored GSH levels. J–N) Western blot analysis of chondrogenic markers (Col2, Aggrecan) and catabolic enzymes (ADAMTS‐5, MMP‐13). Abnormal mechanical stress suppressed anabolic and enhanced catabolic markers, which were reversed by both nanoparticles, with CS‐NAC‐NPs exerting stronger protective effects. Data represent mean ± SD; ^*^ *P* < 0.05, ^**^ *P* < 0.01.

We next evaluated whether the two types of nanoparticles exert protective effects on chondrocytes. First, we determined their optimal working concentration. Under conditions of abnormal mechanical stress (1 MPa) and inflammatory stimulation (TNF‐α, 10 ng mL^−1^), both types of nanoparticles at a concentration of 10 μg mL^−1^ effectively preserved chondrocyte viability (**Figure** **4**A,B). Given that abnormal mechanical stress is a major contributor to chondrocyte injury, we subsequently used mechanical stress as a model to investigate the protective mechanisms of these nanoparticles. As shown in Figure 4C,D, exposure to abnormal mechanical stress significantly increased intracellular ROS levels in chondrocytes. Treatment with either NAC‐NPs or CS‐NAC‐NPs reduced ROS accumulation, with CS‐NAC‐NPs demonstrating a more pronounced effect. Applying nanoparticles alone does not cause any changes in the level of ROS (Figure S6A,B, Supporting Information). Further experiments revealed that mechanical stress impaired mitochondrial activity and membrane potential, while both nanoparticles restored these mitochondrial functions. Notably, CS‐NAC‐NPs induced greater improvements in mitochondrial membrane potential and activity than NAC‐NPs alone (Figure 4E–H). Adding only nanoparticles will not have any effect on the mitochondrial activity of chondrocytes (Figure S6C,D, Supporting Information). Both types of nanoparticles replenished intracellular GSH levels, with CS‐NAC‐NPs achieving a higher degree of restoration (Figure 4I). At the protein level, neither of the two types of nanoparticles alone would cause any changes in the synthesis or metabolic levels (Figure S6E–G, Supporting Information). Abnormal mechanical stress suppressed anabolic markers (Col2, Aggrecan) and upregulated catabolic markers (ADAMTS‐5, MMP‐13), thereby accelerating cartilage matrix degradation. Treatment with either nanoparticle reversed these changes by promoting anabolic marker expression and inhibiting catabolic enzyme production, thus preserving the cartilage matrix. Among the two, CS‐NAC‐NPs conferred stronger protective effects than NAC‐NPs (Figure 4J–N).

### 2.4 CS‐NAC‐NPs Protect Articular Cartilage and Ameliorate OA Progression

**Figure 5:**
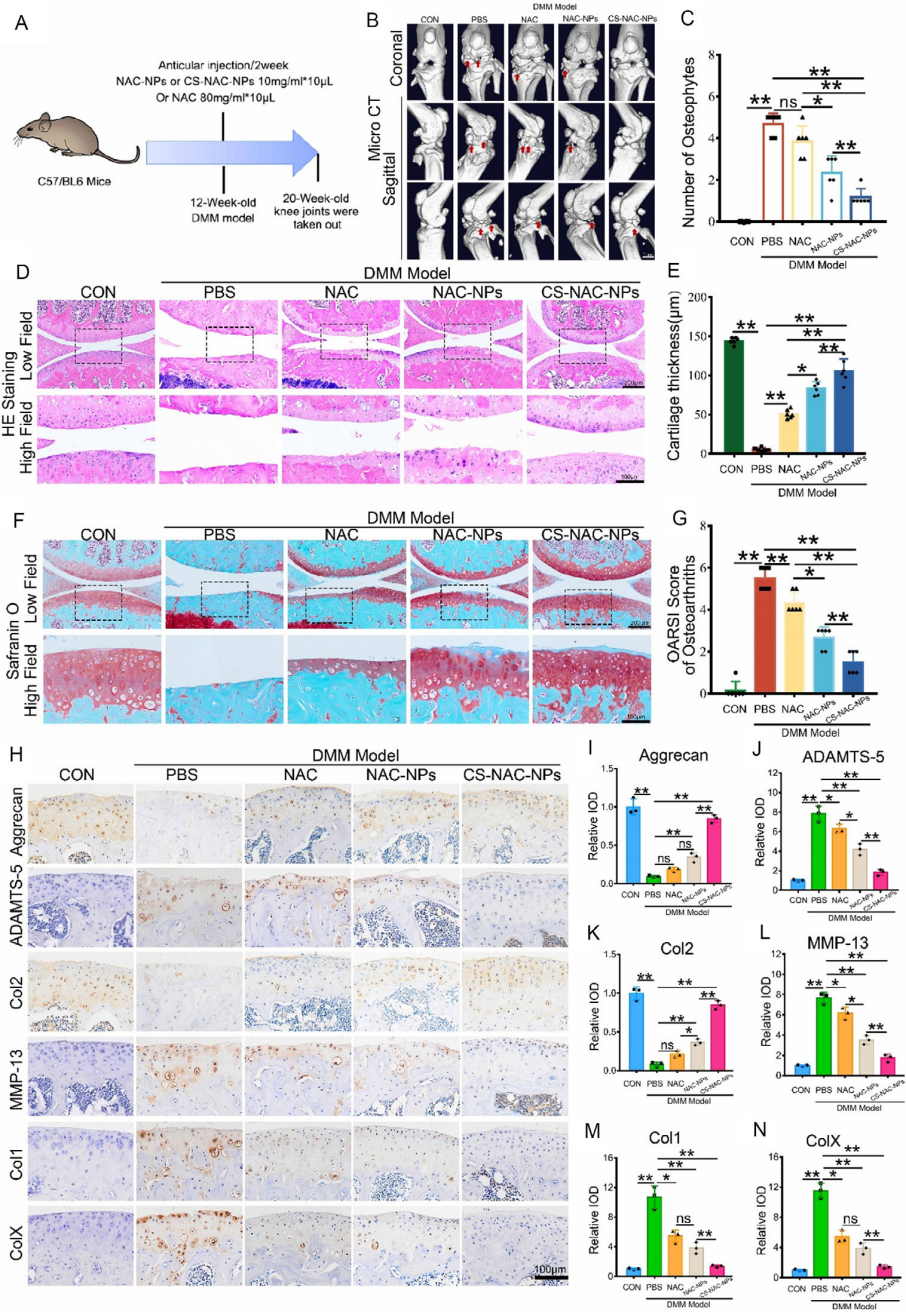
CS‐NAC‐NPs protect articular cartilage and attenuate OA progression in a mouse model. A) Schematic diagram of the experimental design. Mice underwent DMM surgery to induce OA and received intraarticular injections of NAC (80 mg mL^−1^), NAC‐NPs (10 mg mL^−1^), or CS‐NAC‐NPs (10 mg mL^−1^) every two weeks for 8 weeks; control group received PBS. B,C) Micro‐CT analysis of knee joints showing osteophyte formation. Quantification revealed a significant reduction in osteophyte volume in both nanoparticle‐treated groups, with CS‐NAC‐NPs demonstrating the most pronounced effect. Arrows show the formation of osteophytes. Scale bar = 1 mm. D,E) H&E staining of joint sections indicated cartilage thickness loss in the OA group; NAC and NAC‐NPs partially restored cartilage thickness, while CS‐NAC‐NPs showed the greatest preservation. Scale bars, 200 μm (low field), 100 μm (high field). Quantitative analysis to measure the thickness of cartilage tissue. F) Representative images of safranin O fast green staining of the indicated groups. Scale bars, 200 μm (low field), 100 μm (high field). G) Osteoarthritis Research Society International (OARSI) score of OA based on the results of safranin O staining (*n* = 10 for each group). H) Immunohistochemical assay of Aggrecan, Col2, ADAMTS‐5, MMP‐13, Col1 and ColX in articular cartilage of the indicated groups. Scale bars 100 μm. I–N) Quantification of immunohistochemical analysis (*n* = 3 for each group). Data are presented as mean ± SD. ^*^ *P* < 0.05, ^**^ *P* < 0.01.

We evaluated and compared the therapeutic effects of two nanoparticle formulations (NAC‐NPs and CS‐NAC‐NPs) and free NAC on knee OA in mice. Following establishment of the OA model, intraarticular injections of 10 μL of either nanoparticles (10 mg mL^−1^) or NAC (80 mg mL^−1^) were administered biweekly. Control mice received an equal volume of PBS (**Figure** **5**A). After 8 weeks, knee joints were harvested for analysis. Micro‐CT imaging demonstrated that both NAC‐NPs and CS‐NAC‐NPs significantly reduced osteophyte formation, with CS‐NAC‐NPs exhibiting a more pronounced preventive effect. In contrast, free NAC did not show a significant reduction in osteophyte formation compared to PBS (Figure 5B,C). Histological analysis using hematoxylin and eosin (H&E) staining revealed substantial cartilage thinning in the OA model group. Treatment with NAC or NAC‐NPs partially preserved cartilage thickness, while CS‐NAC‐NPs demonstrated the greatest protective effect (Figure 5D,E). Safranin O staining and corresponding OARSI scores further confirmed that all treatments improved cartilage integrity, with CS‐NAC‐NPs providing the most significant reduction in OARSI scores (Figure 5F,G). Immunohistochemical staining showed that OA induction reduced the expression of anabolic markers (Col2 and Aggrecan) and increased the expression of catabolic enzymes (ADAMTS‐5 and MMP‐13). All three treatments effectively restored anabolic marker expression and inhibited catabolic marker expression. Notably, CS‐NAC‐NPs treatment resulted in the most robust upregulation of Col2 and Aggrecan and the greatest suppression of ADAMTS‐5 and MMP‐13 compared to NAC or NAC‐NPs (Figure 5H–N). In addition, CS‐NAC‐NPs significantly reduced the expression levels of Col1 and ColX, thereby suppressing fibrotic tissue proliferation and chondrocyte hypertrophy–associated ossification.

### 2.5 CS‐NAC‐NPs May Regulate GPX4‐Mediated Ferroptosis in Chondrocytes

**Figure 6:**
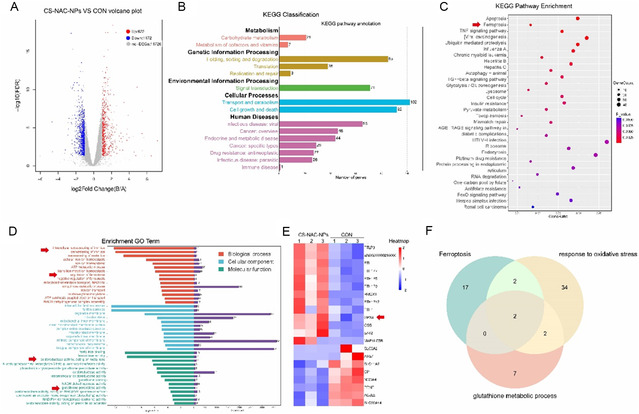
CS‐NAC‐NPs regulate GPX4‐mediated ferroptosis in chondrocytes. A) Volcano plot of differentially expressed genes between CS‐NAC‐NPs–treated and PBS‐treated chondrocytes after 24 h of incubation, with 622 genes upregulated and 1,172 genes downregulated. B) KEGG functional classification of differentially expressed genes. C) KEGG pathway enrichment. D) GO term enrichment. E) Heatmap showing the differentially expressed ferroptosis‐related genes after treatment with CS‐NAC‐NPs. F) Venn diagram illustrating two overlapping genes (*GPX4*, *GSS*) identified among enriched ferroptosis, glutathione metabolic process, and response to oxidative stress‐related pathways.

To elucidate the mechanism by which CS‐NAC‐NPs protect chondrocytes, we performed transcriptomic analysis following a 24‐h co‐incubation of chondrocytes with CS‐NAC‐NPs or an equal volume of PBS. RNA sequencing revealed 622 upregulated and 1,172 downregulated genes (**Figure** **6**A). KEGG classification indicated that the differentially expressed genes were enriched in pathways related to cell death, intracellular transport, and immune system diseases (Figure 6B). Further KEGG pathway enrichment analysis highlighted a strong association with the ferroptosis signaling pathway (Figure 6C). Gene Ontology (GO) term enrichment further showed significant differences in iron ion homeostasis, ferroptosis regulation, oxidative stress response, and GSH metabolism (Figure 6D). Among the genes involved in the ferroptosis regulatory pathway, we identified several differentially expressed genes, including GPX4, a key enzyme known to suppress ferroptosis. Notably, CS‐NAC‐NPs markedly upregulated GPX4 expression (Figure 6E). Cross‐analysis of three enriched processes—ferroptosis, Glutathione metabolic process, and response to oxidative stress—revealed two common differentially expressed genes: GPX4 and GSS. Given that NAC supplementation restores intracellular GSH and possesses potent antioxidant properties, and considering the strong association between CS‐NAC‐NPs and GPX4‐mediated ferroptosis, we propose that CS‐NAC‐NPs may exert their protective effect on chondrocytes by replenishing GSH and regulating GPX4‐dependent ferroptosis.

### 2.6 CS‐NAC‐NPs Fail to Exert Therapeutic Effects in GPX4 Cartilage‐Specific Knockout Mice with Osteoarthritis

**Figure 7:**
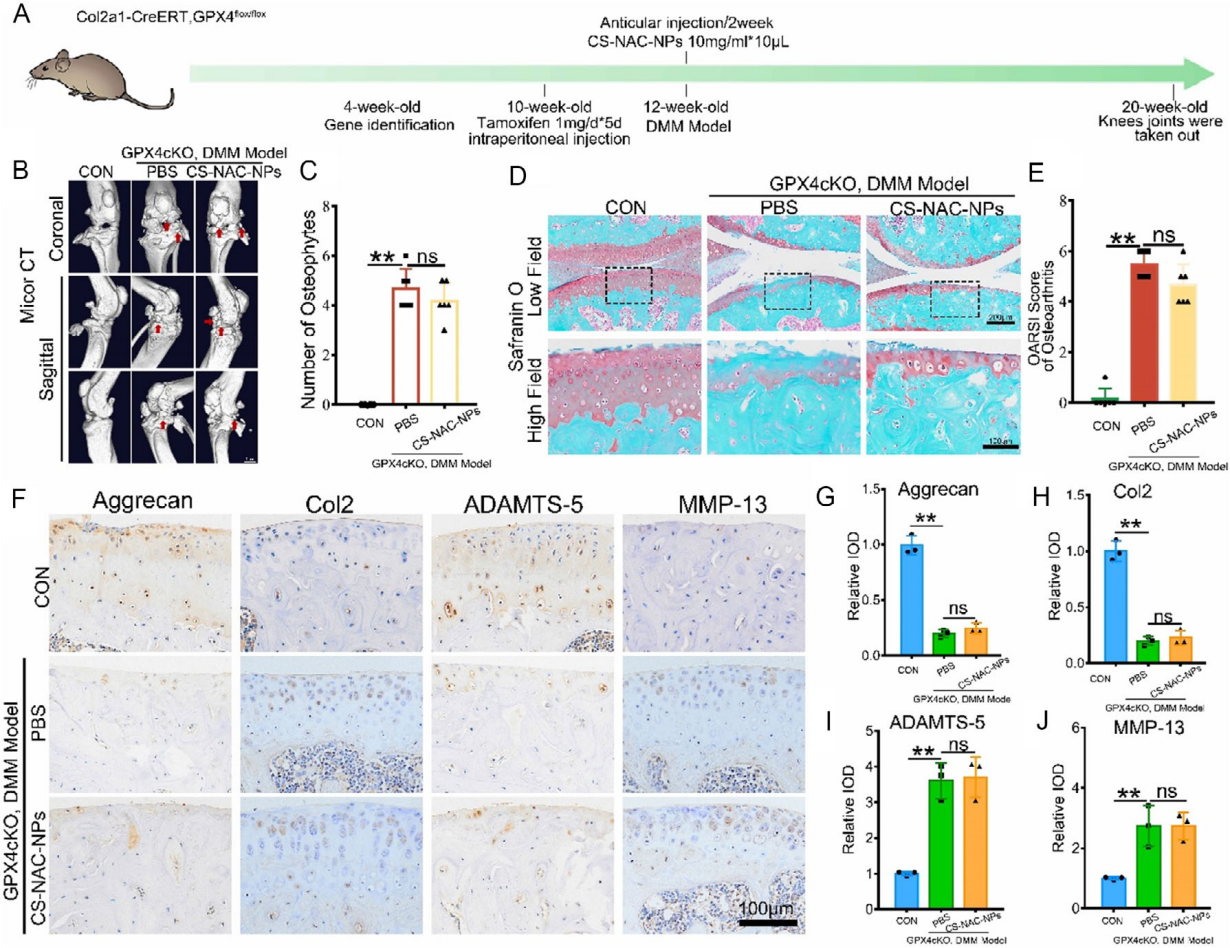
CS‐NAC‐NPs fail to protect articular cartilage in GPX4 cartilage‐specific knockout mice. A) Schematic of experimental design: GPX4cKO mice underwent DMM surgery followed by intraarticular injection of CS‐NAC‐NPs or PBS. B,C) Micro‐CT images (B) and quantitative analysis (C) of osteophyte formation in the knee joints (*n* = 6). Arrows show the formation of osteophytes. Scale bar = 1 mm. D,E) Safranin O–Fast Green staining (D) and OARSI scoring (E) of cartilage damage (*n* = 6). F–J) Immunohistochemical staining and quantification of cartilage matrix synthesis and degradation markers, including Col2, Aggrecan, ADAMTS‐5, and MMP‐13 (*n* = 3). All data are presented as mean ± SD. ns: not significant. ^*^ *P* < 0.05, ^**^ *P* < 0.01.

To determine whether CS‐NAC‐NPs protect articular cartilage by regulating GPX4‐mediated ferroptosis in chondrocytes, we generated cartilage‐specific GPX4 knockout (GPX4cKO) mice and induced osteoarthritis using the destabilization of the medial meniscus (DMM) model, followed by intraarticular injection of CS‐NAC‐NPs (**Figure** **7**A). IF (Figure S7A,B, Supporting Information) and WB (Figure S7C,D, Supporting Information) revealed that GPX4 expression in chondrocytes of mouse knee cartilage was markedly suppressed. Micro‐CT analysis revealed that CS‐NAC‐NPs failed to significantly reduce osteophyte formation in the knee joints of GPX4^cKO mice (Figure 7B,C). Safranin O–Fast Green staining showed that the protective effects of CS‐NAC‐NPs on cartilage tissue were diminished in the absence of GPX4, with no significant reduction in OARSI scores compared to the PBS‐treated group (Figure 7D,E). Furthermore, immunohistochemical analysis of anabolic (Col2, Aggrecan) and catabolic (ADAMTS‐5, MMP‐13) markers indicated that CS‐NAC‐NPs did not significantly enhance cartilage matrix synthesis or inhibit matrix degradation in GPX4‐deficient mice (Figure 7F–J).

### 2.7 CS‐NAC‐NPs Elevate GSH Levels and Protect Chondrocyte Viability by Regulating GPX4‐Mediated Ferroptosis

**Figure 8:**
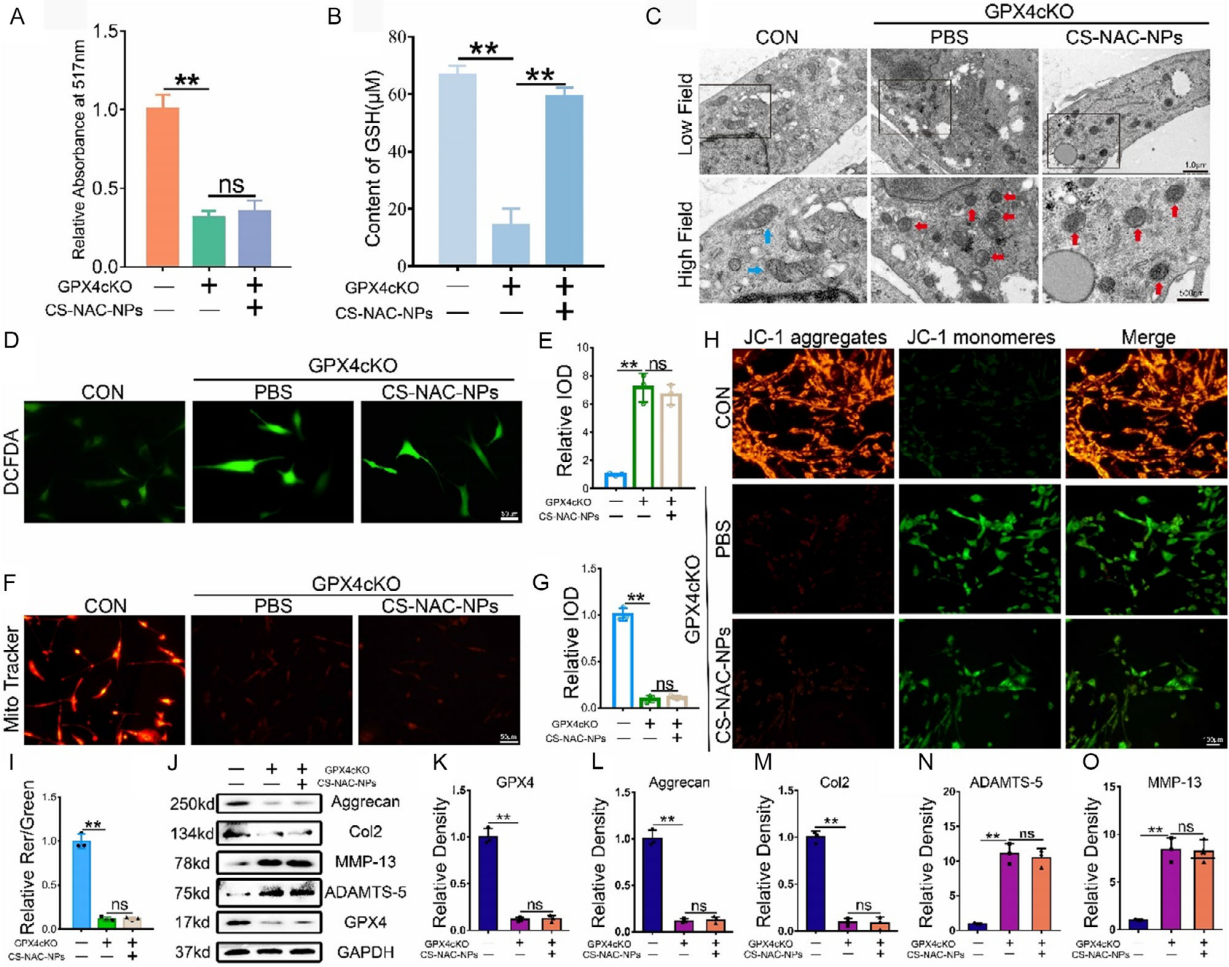
CS‐NAC‐NPs fail to protect chondrocytes from ferroptosis in the absence of GPX4. A) Cell viability of GPX4 cartilage‐specific knockout chondrocytes subjected to 1 MPa abnormal mechanical stress with or without CS‐NAC‐NPs treatment for 24 h, assessed by a Calcein‐AM/PI Viability/Cytotoxicity Assay Kit. B) Intracellular GSH levels measured after CS‐NAC‐NPs treatment in GPX4cKO chondrocytes. C) TEM images showing mitochondrial morphology; ferroptosis features such as condensed mitochondrial membranes and reduced cristae were observed. Blue arrows show the normal mitochondria. Red arrows show the shrunken mitochondria. Scale bars, 1 μm (low field), 500 nm (high field). D) Representative images of ROS levels in chondrocytes. Scale bar = 50 μm. E) Quantitative analysis of fluorescence intensity (*n* = 3 for each group). F) Representative fluorescence images of mitochondria in chondrocytes. Scale bar = 50 μm. G) Quantitative analysis of fluorescence intensity (*n* = 3 for each group). H) Mitochondrial membrane potential was detected by JC‐1 assay. Scale bar = 50 μm. I) The relative IOD ratio of red fluorescence to green fluorescence was used for quantitative analysis (*n* = 3 for each group). J–O) Western blot analysis of GPX4, anabolic markers (Col2, Aggrecan), and catabolic markers (MMP‐13, ADAMTS‐5) expression in chondrocytes after CS‐NAC‐NPs treatment. All data are presented as mean ± SD; ^*^ *p* < 0.05, ^**^ *p* < 0.01, ns: not significant.

In vitro, chondrocytes were isolated from GPX4cKO mice and subjected to abnormal mechanical stress with or without CS‐NAC‐NPs treatment. In the absence of GPX4, CS‐NAC‐NPs failed to maintain chondrocyte viability (**Figure** **8**A). After supplementing with the GPX4 protein, the activity of the chondrocytes was restored (Figure S8A, Supporting Information). Although CS‐NAC‐NPs effectively increased intracellular GSH levels (Figure 8B), the deficiency of GPX4 led to persistent ferroptosis in chondrocytes, as evidenced by mitochondrial morphological changes observed under transmission electron microscopy, characteristic of ferroptosis (Figure 8C). After supplementing with the GPX4 protein, the chondrocytes ferroptosis was inhibited (Figure S8B, Supporting Information). Furthermore, CS‐NAC‐NPs treatment did not significantly reduce intracellular ROS levels compared to the PBS group in GPX4‐deficient cells (Figure 8D,E). Similarly, after supplementing with the GPX4 protein, the level of ROS decreased (Figure S8C,D, Supporting Information). Furthermore, mitochondrial activity and membrane potential were not notably restored in the CS‐NAC‐NPs group compared to the PBS group (Figure 8F–I). After supplementing with the GPX4 protein, the mitochondrial activity was restored (Figure S8E,F, Supporting Information). Western blot analysis confirmed efficient knockdown of GPX4. Compared to PBS treatment, CS‐NAC‐NPs did not significantly upregulate anabolic markers or downregulate catabolic markers in GPX4‐deficient chondrocytes (Figure 8J–O).

## 3 Discussion

Our study introduces a chondrocyte‐targeted nanodelivery system, CS‐NAC‐NPs, designed to restore redox balance via glutathione (GSH) replenishment and regulated glutathione peroxidase 4 (GPX4)‐mediated ferroptosis. Compared with conventional NAC delivery or nontargeted nanocarriers, CS‐NAC‐NPs demonstrated superior intraarticular retention, enhanced cartilage specificity, and greater therapeutic efficacy in mice OA models.

The pathogenesis of OA is multifactorial and involves mechanical, inflammatory, and oxidative stressors that converge on chondrocyte dysfunction and cartilage degradation.^[^ ^19^, ^20^, ^21^, ^22^, ^23^, ^24^ ^]^ In recent years, ferroptosis—a regulated form of iron‐dependent cell death characterized by lipid peroxidation and GSH depletion—has emerged as a key contributor to chondrocyte death and OA progression.^[^ ^9^, ^25^, ^26^, ^27^, ^28^ ^]^ Previous studies, including our own, have demonstrated that excessive mechanical loading activates Piezo1‐mediated calcium influx, mitochondrial dysfunction, and ROS overproduction, which collectively compromise GSH metabolism and induce ferroptosis.^[^ ^6^ ^]^ Therefore, restoring redox balance and inhibiting ferroptosis represents a promising disease‐modifying strategy for OA.

NAC is a well‐established antioxidant that functions both as a direct free radical scavenger and as a precursor for GSH biosynthesis.^[^ ^29^, ^30^ ^]^ However, its clinical application in OA is limited by poor cartilage penetration, rapid synovial clearance, and nonspecific biodistribution. To address these challenges, we utilized a PLGA‐based nanoparticle platform and functionalized its surface with chondroitin sulfate (CS), a cartilage‐binding glycosaminoglycan known to target overexpressed CD44 receptors on chondrocytes and electrostatically bind to type II collagen in the extracellular matrix.^[^ ^17^, ^31^ ^]^ The hydrolytic degradation of PLGA into lactic acid and glycolic acid—two naturally metabolizable byproducts—minimizes systemic toxicity and facilitates sustained drug release in vivo.^[^ ^32^ ^]^ In the context of intraarticular therapy, PLGA nanoparticles offer the additional advantage of enhanced retention within the joint space by resisting rapid synovial clearance, thus extending the local therapeutic window.^[^ ^33^, ^34^ ^]^ This dual‐targeting strategy enabled improved retention and internalization of NAC within cartilage, as evidenced by enhanced fluorescence signals and sustained joint residence of FITC‐labeled CS‐NAC‐NPs up to 14 days postinjection.

Our in vitro assays demonstrated that CS‐NAC‐NPs are biocompatible across a wide range of concentrations and exhibit superior cellular uptake compared to unmodified NAC‐NPs. Under abnormal mechanical stress, CS‐NAC‐NPs more effectively preserved chondrocyte viability, reduced intracellular ROS accumulation, maintained mitochondrial function and membrane potential, and replenished intracellular GSH levels. Notably, they also promoted the expression of anabolic markers (Col2, Aggrecan) and inhibited catabolic enzymes (ADAMTS‐5, MMP‐13), collectively indicating robust protection against mechanical stress‐induced chondrocyte degeneration.

The in vivo efficacy of CS‐NAC‐NPs was demonstrated in a surgically induced OA mouse model, where intraarticular injections significantly reduced osteophyte formation, preserved cartilage thickness, lowered OARSI scores, and improved histological markers of cartilage metabolism. Compared to free NAC and NAC‐NPs, CS‐NAC‐NPs provided the most robust protection, underscoring the importance of both targeted delivery and sustained release in achieving therapeutic efficacy.

Transcriptomic profiling further revealed that CS‐NAC‐NPs upregulated 622 and downregulated 1172 genes in chondrocytes, with KEGG and GO enrichment analyzes identifying ferroptosis, oxidative stress, and GSH metabolism as highly enriched pathways. Of particular importance was the upregulation of GPX4—a central regulator of ferroptosis that reduces lipid peroxides in a GSH‐dependent manner.^[^ ^35^, ^36^ ^]^ GPX4 and GSS were identified as overlapping genes among ferroptosis, oxidative stress response, and glutathione metabolism pathways, reinforcing the hypothesis that CS‐NAC‐NPs exert their protective effects by modulating the GSH‐GPX4‐Ferroptosis axis.

To confirm that GPX4 is required for the observed therapeutic benefits, we employed GPX4 cartilage‐specific knockout (GPX4cKO) mice. In these models, CS‐NAC‐NPs failed to reduce osteophyte formation, restore cartilage matrix, or modulate anabolic/catabolic markers, indicating that the protective effects of CS‐NAC‐NPs are indeed dependent on the presence of functional GPX4. In vitro assays using GPX4‐deficient chondrocytes corroborated these findings. Although CS‐NAC‐NPs elevated intracellular GSH levels, ferroptosis‐associated mitochondrial abnormalities and ROS accumulation persisted, and no significant improvement in chondrogenic markers was observed. These results collectively demonstrate that CS‐NAC‐NPs mediate their antiferroptotic effects primarily through GPX4‐dependent mechanisms.

There are several notable implications of our findings. First, this study highlights the feasibility and advantage of integrating mechano‐targeting principles into drug delivery platforms for OA therapy. Our CS‐conjugated nanoparticles achieved enhanced joint localization and cellular uptake. Second, the work provides compelling evidence for ferroptosis as a therapeutically targetable pathway in OA and supports GPX4 as a crucial molecular checkpoint in cartilage homeostasis. Third, our approach of utilizing a biodegradable and clinically translatable material (PLGA) paves the way for potential clinical development of NAC‐based nano formulations.

Nonetheless, several limitations warrant further investigation. Although we confirmed GPX4‐dependency of CS‐NAC‐NPs in murine models, human cartilage exhibits species‐specific differences in ECM composition and chondrocyte biology that may affect therapeutic translation. Additionally, while CS enhanced chondrocyte targeting, future studies could incorporate more specific ligands (e.g., anti‐CD44 antibodies or collagen‐binding domains) to further improve precision delivery. Finally, long‐term efficacy, repeated dosing, and immunogenicity studies are required before advancing toward clinical application.

**Figure 9:**
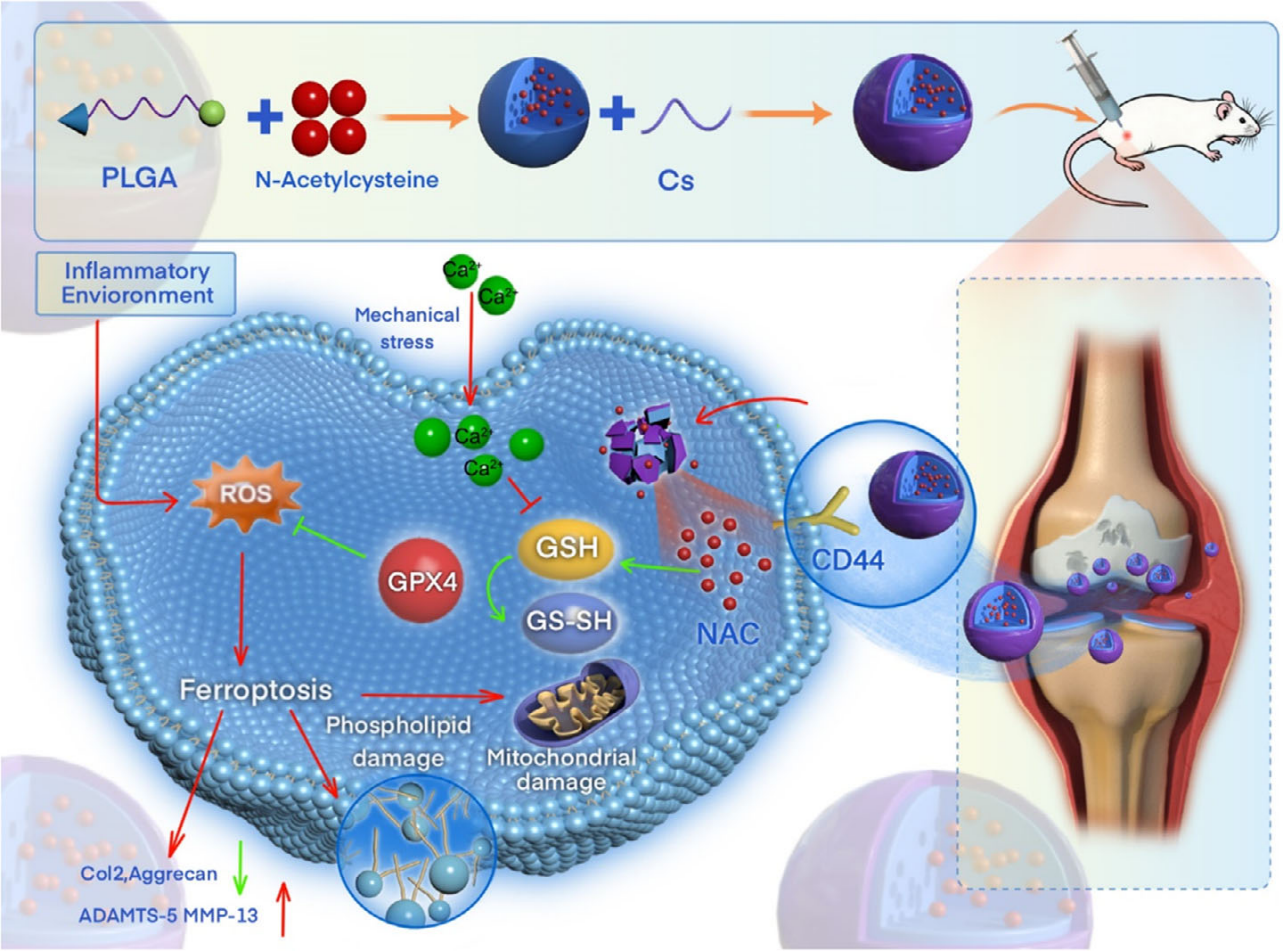
The schematic diagram of the mode of action for CS‐NAC‐NPs in protecting articular cartilage and treating osteoarthritis.

In conclusion, our study presents a novel nanotherapeutic strategy that integrates redox modulation and biomechanical targeting to inhibit chondrocyte ferroptosis and preserve cartilage integrity in OA. CS‐NAC‐NPs significantly outperformed free NAC and unmodified nanoparticles by sustaining intraarticular drug levels, targeting stressed chondrocytes, and reactivating GPX4‐mediated antioxidative defenses (**Figure** **9**). These findings establish a strong foundation for the continued development of ferroptosis‐targeted, biomaterial‐enabled therapies in osteoarthritis and other degenerative joint diseases.

## 4 Experimental Section

### Materials

Poly (lactic‐co‐glycolic acid) (PLGA), *N*‐Acetylcysteine (NAC), 1‐ethyl‐3‐(3‐dimethylaminopropyl) carbodiimide (EDC) and Dichloromethane were purchased from MACKLIN. Chondroitin sulfate (CS) was purchased from Solarbio. *N*‐hydroxysuccinimide (NHS) was purchased from Aladdin. Dialysis bags were purchased from Beyotime (China). Tween 80 was purchased from Sinopharm Chemical Reagent (China).

### Fabrication of NAC‐NPs

NAC‐NPs were synthesized utilizing a double emulsion (W/O/W) solvent evaporation technique. Briefly, 30 mg of NAC was dissolved in 1 mL of distilled water, while 150 mg of PLGA was solubilized in 3 mL of dichloromethane. These solutions were emulsified under intermittent sonication (5 min) to form a primary water‐in‐oil (W/O) emulsion. Subsequently, 5 mL of a 0.1% w/v polyvinyl alcohol (PVA) aqueous solution was incorporated into the primary W/O emulsion. This mixture was subjected to further intermittent sonication (5 min), facilitating the formation of a stable double emulsion (W/O/W). The resultant emulsion was then agitated vigorously under reduced pressure for 4 h to ensure complete evaporation of the organic phase. The synthesized NAC‐NPs were recovered via centrifugation (13 000 × g, 15 min), washed, and resuspended. Finally, the purified nanoparticles underwent lyophilization and were stored at −20 °C for subsequent use.

### Surface Functionalization of NPs with CS

Following the 4‐h vigorous agitation under reduced pressure, the purified NAC‐NPs were harvested from the W/O/W emulsion via centrifugation and resuspension. The recovered nanoparticles were then dispersed in an acetate buffer solution (sodium acetate/acetic acid, pH 6.0) and chemically activated through reaction with 1‐ethyl‐3‐(3‐dimethylaminopropyl) carbodiimide (EDC) and *N*‐hydroxysuccinimide (NHS) for 1 h. Subsequently, the activated CS solution was introduced to the NAC‐NPs suspension, and the conjugation reaction was allowed to proceed at ambient temperature. After a 3‐h incubation period, the resultant CS‐NAC‐NPs conjugates were collected by centrifugation (13 000 × g, 15 min), resuspended, lyophilized, and archived at −20 °C for long‐term storage.

### Measurements

Size distribution and zeta potential of NPs was measured by Malvern dynamic laser scattering (DLS) analyzer (NanoZS, UK). Transmission electron microscope (TEM) images of the NPs were acquired using ZESSI GeminiSEM500 (German). Fourier transforms infrared spectroscopy(FTIR) was measured by Tensor II Infrared Spectrometer (Bruker, USA).

### Multibioresponsive Release Profiles of NPs

Dialysis method was used to investigate the controlled release properties of the nanoparticle drug. Briefly, the CS surface‐functionalized nanoparticles loaded with NAC (CS‐NAC‐NPs) were fully suspended in deionized water (equivalent to 200 μg NAC). The suspension was then transferred into dialysis bags (MWCO = 1400 Da), and the ends of the bags were sealed. These bags were subsequently immersed in test tubes containing release buffers of different compositions. The tubes were placed in a water bath or orbital shaker set at 37 °C with a shaking speed of 120 rpm. At predetermined time intervals, a suitable volume (≈2 mL) of the release buffer was withdrawn for drug concentration analysis, and fresh release buffer was added to maintain the volume in the test tubes. The quantification of NAC released into the medium was performed using a fluorescence spectrophotometer (Model RF‐600, Shimadzu Corporation, Japan).

### Animals

All experimental procedures involving animals in this study were performed in strict compliance with institutional guidelines and were approved by the Animal Ethics Committee of Qilu Hospital, Shandong University (Approval No. KYLL‐2024(ZM)‐1028). C57BL/6 wild‐type (WT) mice, sourced from the Experimental Animal Center of Shandong University, were utilized in the experiments.

Eight‐week‐old mice were randomly allocated into two experimental cohorts (*n* = 30 per group). Each cohort received an intraarticular injection into the left knee joint with either FITC‐conjugated CS‐NAC‐NPs or FITC alone. In vivo fluorescence imaging was performed longitudinally at the following postinjection intervals: Day 0 (day of injection), Day 3, Day 7, Day 14, and Day 30. On each designated imaging day, a subset of three animals per cohort was randomly selected, euthanized under pentobarbital anesthesia (100 mg kg^−1^), and subjected to tissue harvest. Specimens collected included the heart, liver, spleen, lung, kidney, and the injected left knee joint. Harvested tissues underwent ex vivo fluorescence quantification and were subsequently fixed and processed for histological sectioning.

To comparatively evaluate the therapeutic efficacy of nanoparticles in OA, twelve‐week‐old murine subjects underwent surgical induction of an OA model via destabilization of the medial meniscus (DMM) in the left knee joint. Commencing at model induction, intraarticular administrations (10 μL) of either NAC (80 mg mL^−1^), NAC‐NPs or CS‐NAC‐NPs (10 mg mL^−1^) were delivered biweekly into the ipsilateral joint cavity. Terminal tissue harvest was performed at the experimental endpoint (8 weeks postmodel induction), wherein knee joint specimens were collected for subsequent comprehensive analyzes.

To elucidate whether the therapeutic effects of CS‐NAC‐NPs are mediated through the GPX4‐regulated ferroptosis pathway, genetically engineered mice with chondrocyte‐specific conditional knockout (cKO) of GPX4 (Col2a1‐CreERT GPX4^flox/flox^) were employed. The breeding strategy, genotyping protocols, and induction regimen for these murine models have been comprehensively delineated in two of our previously published manuscripts.^[^ ^6^, ^37^ ^]^ At 12 weeks of age, GPX4 cKO mice underwent surgical induction of an OA model via destabilization of the medial meniscus (DMM) in the left knee joint. Concomitant with model establishment, mice received intraarticular injections (10 μL) of either CS‐NAC‐NPs (10 mg mL^−1^) or phosphate‐buffered saline (PBS); vehicle control administered biweekly into the ipsilateral joint. Terminal harvest of knee joint tissues was performed at the experimental endpoint (8 weeks postmodel induction) for subsequent examinations.

Knee joints were harvested from all mice eight weeks postsurgical induction of the DMM model. Animals presenting with infections, tumors, or compromised health status were excluded from subsequent analyzes. To compensate for potential attrition due to infection, an additional three to four mice per group were included as a safeguard.

For each experimental condition, a minimum of three biological replicates (mice) were utilized. Data are presented as mean ± standard deviation (SD). Statistical comparisons between groups were performed using one‐way analysis of variance (ANOVA) implemented in GraphPad Prism 7 software (GraphPad Software Inc., San Diego, CA, USA).

### Primary Cell Isolation and Culture

Articular cartilage tissues from the distal femur and proximal tibia of 5‐day‐old mice were meticulously dissected under microscopic guidance. Following isolation, the tissues underwent enzymatic digestion using 0.2% type II collagenase (Gibco) at 37 °C for 8 h. The isolated chondrocytes were then seeded at a density of 5.7 × 10^5^ cells cm^−^^2^ and maintained in DMEM/F12 growth medium (HyClone, Logan, USA) containing 10% fetal bovine serum (FBS); Gibco, USA, 100 U mL^−1^ penicillin, and 0.1 mg mL^−1^ streptomycin (HyClone, USA), under standard culture conditions (37 °C, 5% CO_2_). Culture medium was refreshed every third day. For all subsequent in vitro experiments, chondrocytes within the first five passages were utilized. Each experiment was conducted with duplicate wells.

GPX4 knockdown in chondrocytes: Primary chondrocytes were isolated from 5‐day‐old Col2a1‐CreERT GPX4^flox/flox^ mice using the aforementioned protocol. To induce conditional knockout of GPX4, the chondrocytes were treated with 10 nM 4‐hydroxytamoxifen (4‐OHT; Apexbio, USA, Cat#B5421) for a period of 3 days.

### In vitro Mechanical Stress Culture Model

The custom‐built apparatus for applying mechanical pressure to cells in vitro was conceptualized based on established methodologies described in the literature.^[^ ^38^, ^39^ ^]^ Comprehensive schematics and step‐by‐step operational protocols are available in our prior publication.^[^ ^6^ ^]^ Briefly, the experimental workflow involved the following steps:

Chondrocytes were initially plated onto glass slides (14 mm or 24 mm diameter). These cell‐laden slides were subsequently mounted onto a dedicated scaffold assembly. The entire assembly, comprising the slides and scaffold, was then enclosed within a pressure chamber filled with complete culture medium. Hydrostatic pressure within the sealed chamber was generated by applying force to a deformable membrane using a pneumatic actuator (FESTO, Germany). Chondrocytes were exposed to cyclic mechanical loading at 1 MPa (1 Hz frequency) for 1 h. This pressure magnitude was selected as it exceeds the ≈0.4 MPa average load reported for murine knee cartilage. To ensure optimal cellular health prior to loading, the assembled chamber containing medium and cells was equilibrated within a standard cell culture incubator for 6 h. Following the pressurization period, either the treated cells (on slides) or femoral head explants were immediately transferred to fresh complete medium for subsequent assays.

### Cell Viability

Cell survival rates were quantified utilizing a Calcein‐AM/PI Viability/Cytotoxicity Assay Kit (Beyotime Biotechnology, China, Cat# C2015). After exposure to the designated experimental conditions for 24 h, cells were stained with the Calcein‐AM/PI working solution and incubated at 37 °C for 30 min. Viable cells were identified by the characteristic green fluorescence emission resulting from intracellular Calcein‐AM conversion. Cellular viability was determined through quantification of fluorescence intensity at 517 nm utilizing a fluorescence spectrophotometer (Model RF‐600, Shimadzu Corporation, Japan).

### Total ROS Measurement

Posttreatment, cells were loaded with 10 μM 2′,7′‐dichlorodihydrofluorescein diacetate (DCFH‐DA; Beyotime Biotechnology, China, Cat# S0033S) and incubated for 30 min. Subsequently, cells were rinsed twice with PBS to remove unincorporated probe. Following trypsinization, the labeled cells were resuspended in PBS supplemented with 5% FBS. The oxidation of nonfluorescent DCFH‐DA by intracellular ROS yields the highly fluorescent derivative 2′,7′‐dichlorofluorescein (DCF). This DCF fluorescence signal, reflecting cellular ROS levels, was quantified using a ZEISS LSM780 confocal laser scanning microscope (ZEISS, Germany). Enhanced green fluorescence intensity served as an indicator of elevated intracellular ROS.

### Analysis of GSH Level

Chondrocytes were exposed to the defined experimental stimuli. Twenty‐four hours poststimulation, cellular lysates were prepared from the relevant experimental groups. GSH concentrations in these lysates were measured employing a Total Glutathione Peroxidase Assay Kit (Beyotime Biotechnology, China, Cat# S0058), strictly adhering to the manufacturer's instructions as outlined previously. Fluorescence or absorbance readings were collected using a Varioskan Flash multimode microplate reader (Thermo Fisher Scientific, Waltham, MA, USA). Cytokine concentrations (μM) were calculated by interpolation from a standard curve constructed using CELLQUEST software.

### JC‐1 Assay

Chondrocytes were treated according to the established experimental conditions. Twenty‐four hours posttreatment, mitochondrial membrane potential was evaluated using the fluorescent probe JC‐1 (Beyotime Biotechnology, China, Cat# C2006), strictly following the manufacturer's protocol. Briefly, stimulated chondrocytes were incubated with JC‐1 staining solution at 37 °C for 20 min. Under conditions of high mitochondrial membrane potential, JC‐1 accumulates within mitochondria, forming aggregates that exhibit red fluorescence emission. Conversely, dissipation of mitochondrial membrane potential promotes the monomeric form of JC‐1, characterized by green fluorescence. Cellular fluorescence was visualized and captured using a ZEISS LSM780 confocal laser scanning microscope (ZEISS, Germany). Alterations in mitochondrial membrane potential were quantified by calculating the ratio of red‐to‐green fluorescence intensity.

### MitoTracker Assay

Chondrocytes were exposed to the designated experimental regimens. Twenty‐four hours posttreatment, the fluorescent probe MitoTracker Red CMXRos (Beyotime Biotechnology, China, Cat# C1035) was employed to label metabolically active mitochondria. Following the prescribed stimulation, cells were incubated with the MitoTracker solution at 37 °C for 30 min. Live‐cell imaging was performed using a ZEISS LSM780 confocal laser scanning microscope (ZEISS, Germany) to capture fluorescence signals. The intensity of the red fluorescence signal correlated with the degree of mitochondrial functional activity.

### Total Protein Extraction and Western Blotting

Chondrocytes were exposed to the designated experimental conditions. Following either immediate processing or a 24‐h incubation period, cellular proteins were extracted using ice‐cold RIPA lysis buffer (Millipore, Billerica, USA) supplemented with 1 mM PMSF (Beyotime Biotechnology, China) for 30 min on ice. The resulting lysates were centrifuged at 12 000 × g for 15 min at 4 °C, and the clarified supernatants were harvested. Protein concentration normalization across all samples was achieved using a BCA protein assay kit (Biotechnology Co, China). Subsequently, equal protein amounts per sample were resolved by electrophoresis on 10% SDS‐polyacrylamide gels (SDS‐PAGE) and electrophoretically transferred onto polyvinylidene difluoride (PVDF) membranes (Millipore, USA). Membranes were blocked for 1 h at ambient temperature in Tris‐buffered saline containing 0.1% Tween‐20 (TBST) and 5% nonfat dry milk. Blocked membranes were then probed overnight at 4 °C with the indicated primary rabbit polyclonal antibodies: anti‐CD44 (1:1000, Abcam, USA), anti‐GPX4 (1:1000, Abcam, USA), anti‐Aggrecan (1:1000, Proteintech, USA), anti‐Col2 (1:1000, Proteintech, USA), anti‐ADAMTS‐5 (1:1000, Abcam, USA), anti‐MMP‐13 (1:1000, Abcam, USA), and anti‐GAPDH‐HRP (1:5000, Proteintech, USA). After extensive washing with TBST, membranes were incubated for 1 h at room temperature with a horseradish peroxidase (HRP)‐conjugated goat antirabbit IgG secondary antibody (1:5000, Jackson ImmunoResearch, USA). Immunoreactive bands were detected employing an Amersham Imager 600 chemiluminescence detection system (GE Healthcare, USA) and quantified densitometrically using Image‐Pro Plus software (v6.0, Media Cybernetics, Inc., USA).

### Micro‐Computed Tomography (Micro‐CT) Analysis

Image acquisition was performed employing an isotropic voxel size of 15 μm. The X‐ray source was operated at 70 kV and 200 μA. Vertebral microarchitecture was examined using a Quantum GX2 micro‐CT imaging system (PerkinElmer, USA). To preserve tissue integrity for subsequent imaging and histological assessment, specimens were initially fixed in paraformaldehyde. For 3D reconstruction and quantitative assessment of sample morphology, scanned datasets from all groups underwent processing with a uniform grayscale threshold prior to morphometric parameter analysis.

### Immunohistochemistry

Knee joint tissues were collected at the 8‐week postoperative time point following DMM model induction. Samples underwent fixation in 4% paraformaldehyde and subsequent decalcification in a 10% ethylenediaminetetraacetic acid (EDTA) solution. Tissues were then embedded in paraffin and sectioned at a thickness of 5 μm. Similarly, human cartilage specimens were paraffin‐embedded and sectioned into 5 μm slices. Following deparaffinization with xylene and graded ethanol series, sections underwent antigen retrieval using citrate buffer (pH 6.0). Nonspecific binding sites were blocked with Bovine Serum Albumin (BSA). Primary antibody incubation was performed overnight at 4 °C using the following: rabbit anti‐ADAMTS‐5 (Abcam, USA; 1:200), rabbit anti‐MMP‐13 (Abcam, USA; 1:200), rabbit anti‐Col1 (Abcam, USA; 1:200), rabbit anti‐ColX (Proteintech, USA; 1:200), rabbit anti‐Aggrecan (Abcam, USA; 1:500), and rabbit anti‐Col2 (Proteintech, USA; 1:200). Subsequently, sections were incubated for 60 min at room temperature with a horseradish peroxidase (HRP)‐conjugated goat antirabbit IgG secondary antibody (Jackson ImmunoResearch, USA; 1:200). Stained sections were examined under an IX71‐SIF microscope (Olympus, Tokyo, Japan), with positive immunoreactivity visualized as brown deposits. Quantification of staining was performed using Image‐Pro Plus 6.0 software (Media Cybernetics, Inc., USA).

Safranin O/Fast Green staining was conducted according to the manufacturer's instructions using a modified kit (Solarbio, Beijing, China). For Hematoxylin and Eosin (H&E) staining, deparaffinized and rehydrated sections were processed using an H&E Staining Kit (Solarbio, Beijing, China), adhering to the standard protocol

### Histopathologic and Quantificational Evaluation of OA

The Osteoarthritis Research Society International (OARSI) histological scoring system was employed to assess the proteoglycan content in articular cartilage, based on Safranin O staining^[^ ^40^ ^]^ (Table S2, Supporting Information).

### Microarray RNA‐Sequence

The chondrocytes were incubated together with the CS‐NAC‐NPs (10 μg mL^−1^), while the control group was added with an equal amount of PBS. Following the specified stimulation protocols, chondrocytes were harvested at the 24‐h time point using a cell scraper. Each sample contained a minimum of 6 × 10^5^ cells. After centrifugation, the resulting cell pellets were collected, rapidly frozen in liquid nitrogen, and maintained on dry ice during transit. Similarly, human knee cartilage specimens were preserved in RNA keeper (Vazyme, Nanjing, China) and shipped on dry ice. Transcriptomic Analysis: Comprehensive gene expression profiling was outsourced to Qinglian Biotech (Beijing, China). To elucidate the associations between mechanical stress and OA pathogenesis, bioinformatic analyzes were performed utilizing the Kyoto Encyclopedia of Genes and Genomes (KEGG) pathway database and Gene Ontology (GO) term annotations.

### TEM

Chondrocytes underwent stimulation as per the designated protocol. At the 24‐h endpoint, cells were detached using a cell scraper, ensuring a minimum yield of 6 × 10^5^ cells per sample. Harvested chondrocytes and human knee cartilage specimens were immediately treated with TEM fixative at 4 °C. All samples were centrifuged and subjected to three washes with 0.1 M phosphate buffer (pH 7.4). A supporting matrix of 1% agarose was prepared through heating and dissolution prior to use. Samples, including human cartilage tissues, were then immersed in a postfixation solution containing 1% osmium tetroxide (OsO_4_) in 0.1 M phosphate buffer (pH 7.4) for 2 h at room temperature. Following postfixation, dehydration was carried out at room temperature using an ascending ethanol series. Samples were subsequently infiltrated with Embed 812 epoxy resin (SPI Supplies, Cat# 90529‐77‐4) and embedded overnight at 37 °C. Embedded samples within molds underwent thermal polymerization at 65 °C for a duration exceeding 48 h. The resulting resin blocks were ultrathin‐sectioned to 60–80 nm thickness using a Leica UC7 ultramicrotome (Leica Microsystems). Sections were contrasted using a saturated alcoholic solution of 2% uranyl acetate followed by 2.6% lead citrate. Finally, cellular ultrastructure was assessed using a Hitachi HT7700 TEM (Hitachi High‐Technologies, Tokyo, Japan), and representative micrographs were acquired.

### Ethics Statement

Animal experiments conducted as part of this research followed institutional guidelines and were approved by the Laboratory Animal Center of Qilu Hospital, Shandong University (Approval No. KYLL‐2024(ZM)‐1028).

### Statistical Analyzes

All experimental data underwent statistical analysis utilizing GraphPad Prism software (version 7, GraphPad Software Inc., San Diego, CA, USA). Depending on the experimental design and the number of groups being compared, appropriate statistical tests were applied: either Student's t‐tests (for two‐group comparisons) or one‐way/two‐way analysis of variance (ANOVA) (for multiple group comparisons). Quantitative results are expressed as mean ± standard deviation (SD). Differences were considered statistically significant when the probability value (*P*) was less than 0.05. To enhance data reliability, all in vitro cellular assays were independently replicated at least three times.

## Abbreviations

**Table:** 

GPX4	Glutathione peroxidase 4
OA	osteoarthritis
GSH	glutathione
DMM	Destabilization of the medial meniscus
MMP‐13	Matrix metallopeptidase 13
ADAMTS‐5	A disintegrin and metalloproteinase with thrombospondin motifs‐5
Col2	Collagen II
ROS	Reactive oxygen species
OARSI	Osteoarthritis Research Society International

## Supporting Information

Supporting Information is available from the Wiley Online Library or from the author.

## Conflict of Interest

The authors declare no conflict of interest.

## Supporting information

Supplementary Material

## Data Availability

The data that support the findings of this study are available from the corresponding author upon reasonable request.
