# Supplementary material for: Chondrocyte‐Targeted Nanoparticles Loaded with N‐Acetylcysteine Protect Articular Cartilage and Attenuate Osteoarthritis by Inhibiting Ferroptosis via Glutathione Maintenance
Source: Small Sci. 2025 Nov 28;6(1):e202500440. doi: 10.1002/smsc.202500440 (PMC12798779; doi:10.1002/smsc.202500440)
Supplement: Supplementary file 1 — Supplementary Material [file SMSC-6-e202500440-s001.pdf]

Chondrocyte-Targeted Nanoparticles Loaded with N-Acetylcysteine Protect Articular Cartilage and Attenuate Osteoarthritis by Inhibiting Ferroptosis via Glutathione Maintenance

Shaoyi Wang[1,2], Fujian Zhang[1], Xiaocong Zhou[3], Jie Yang[1], Zhe Li[1], Songlin Li[1], Qunshan Lu[1, #], Houyi Sun[1, #], Peilai Liu[1, #].

Affiliations:

1. Department of Orthopaedic Surgery, Qilu Hospital, Cheeloo College of Medicine, Shandong University, Jinan, Shandong, 250012, P. R. China.

2. Qilu Hospital of Shandong University Spine and Spinal Cord Disease Research Center- ICMRS Collaborating Center for Orthopaedic translational Research, Shandong University, Jinan, Shandong, 250012, P.R. China.

3. Health Management Centre, The First Affiliated Hospital of Shandong First Medical University, Jinan, Shandong, 250012, P. R. China.

#To whom correspondence should be addressed:

Qunshan Lu, Department of Orthopaedic Surgery, Qilu Hospital, Cheeloo College of Medicine, Shandong University, 107 Wenhuaxi Road, Jinan, P. R. China 250012. Email: luqunshan@qiluhospital.com

Houyi Sun, Department of Orthopaedic Surgery, Qilu Hospital, Cheeloo College of Medicine, Shandong University, 107 Wenhuaxi Road, Jinan, P. R. China 250012.

Email: 15051514605@163.com

Peilai Liu, Department of Orthopaedic Surgery, Qilu Hospital, Cheeloo College of Medicine, Shandong University, 107 Wenhuaxi Road, Jinan, P. R. China 250012. Email: 199362000205@email.sdu.edu.cn

Supplementary Materials and Method

Entrapment efficiency and drug loading

The Encapsulation efficiency (EE) and drug loading (DL) were determined using an indirect method based on ultracentrifugation. Following the preparation of the double emulsion, the resulting nanoparticle suspension was centrifuged at 4500 rpm for 40 minutes (Centrifuge 5418R, Eppendorf, USA). The concentration of unencapsulated drug in the supernatant was then quantified at 204 nm using a fluorescence spectrophotometer (Model RF-600, Shimadzu Corporation, Japan). EE was calculated as the ratio of the amount of NAC encapsulated within the nanoparticles to the total amount of NAC initially added. DL was defined as the amount of NAC encapsulated divided by the total weight of the formulation, including both NAC and PLGA. The percentages of EE and DL were calculated using the following formulas:

$$\%\text{Encapsulation efficiency (EE)} = \frac{\text{Total drug added} - \text{Amount of free drug}}{\text{Total drug added}} \times 100$$

$$\text{Drug Loading } (\mu\text{g/mg}) = \frac{\text{Amount of drug added} - \text{Unentrapped drug}}{\text{Weight of nanoparticles recovered}} \times 100$$

GPX4 protein rescue experiment

Chondrocytes with GPX4 knockout were treated with CS-NAC-NPs and subjected to 1 MPa mechanical compression for 24 hours, with or without the addition of exogenous GPX4 protein. Normal chondrocytes without any treatment served as the control group. After 24 hours, cell viability was assessed using live/dead staining, mitochondrial morphology was examined by TEM, intracellular ROS levels were determined by ROS staining, and mitochondrial activity was evaluated using

MitoTracker staining.

Knockdown of CD44 by siRNA

To knock down CD44, chondrocytes were transfected with 100nM siCD44(sc-35534; Santa Cruz Biotechnology) and scrambled control siRNA (scRNAi, sc-37007; Santa Cruz Biotechnology) by using the siRNA transfection reagent PROTPCOL (Polyplus, France). After 3 days, Transfection efficiency was assessed by IF and WB. After incubation with siTHBS1 or scRNAi for 3 days, chondrocytes were treated.

Immunofluorescence Staining

Cells were processed according to the experimental protocol and fixed with 4% paraformaldehyde either immediately or after a 24-hour incubation period. Subsequently, they were permeabilized in 0.2% Triton X-100 for 20 minutes and then blocked with 1% bovine serum albumin (BSA) for 30 minutes to reduce nonspecific binding. The samples were next incubated overnight at 4°C with a primary antibody against CD44 (rabbit anti-CD44, 1:200, Abcam, USA). On the following day, the cells were exposed to a fluorescently conjugated secondary antibody (goat anti-rabbit IgG, 1:100, Abbkine, China) for 1 hour at room temperature. Fluorescence images were obtained using an IX71-SIF inverted microscope (Olympus, Tokyo, Japan) and quantitatively analyzed with Image-Pro Plus 6.0 software (Media Cybernetics, Inc., USA).

The immunofluorescence staining of tissue sections was conducted following procedures similar to those used in immunohistochemistry. Briefly, the sections were

incubated overnight at 4 °C with a rabbit anti-GPX4 primary antibody (1:100, Abcam, USA). After washing, they were treated with a fluorescently labeled goat anti-rabbit IgG secondary antibody (1:100, A23320, Abbkine, China) for 1 hour at room temperature. Fluorescent images were captured using an IX71-SIF fluorescence microscope (Olympus, Tokyo, Japan) and quantitatively analyzed with Image-Pro Plus 6.0 software (Media Cybernetics, Inc., USA).

Table S1: Physicochemical characteristic of NPs fabricated by NAC and PLGA with various concentrations

NPs	NAC solution (mg/mL)	PLGA solution (mg/mL)	Particle size (nm)	Zeta potential (mV)	Loading amount (%)	Encapsulation efficiency (%)
NPs-1	5	25	160	-11.5	1.2	35.7
NPs-2	15	25	186	-12.0	2.3	39.0
NPs-3	30	25	188	-12.7	4.9	58.9
NPs-4	60	25	190	-13.5	4.0	61.4
NPs-5	30	5	203	-10.5	1.9	30.6
NPs-6	30	25	253	-11.5	2.7	33.6
NPs-7	30	50	277	-12.6	5.6	55.6
NPs-8	30	75	450	-25.6	3.0	40.5

Table S2 Physicochemical characteristic of NPs(n=3)

	Particle size (nm)	Zeta potential (mV)
NAC-NPs	277.7 ±12.55	-12.6 ± 1.48
CS-CAC-NPs	372.3 ± 13.04	-19.33 ± 0.57

Table S3 Murine OARSI score system

Grade	Osteoarthritic damage
0	Normal cartilage without damage
0.5	Loss of Safranin-O staining while no detectable structural change
1	Small fibrillation
2	Vertical damage of cartilage limited to superficial layer
3	Vertical damage, no more than 25% of the cartilage surface
4	Vertical damage, 25-50% of the cartilage surface
5	Vertical damage, 50-75% of the cartilage surface
6	Vertical damage, more than 75% of the cartilage surface

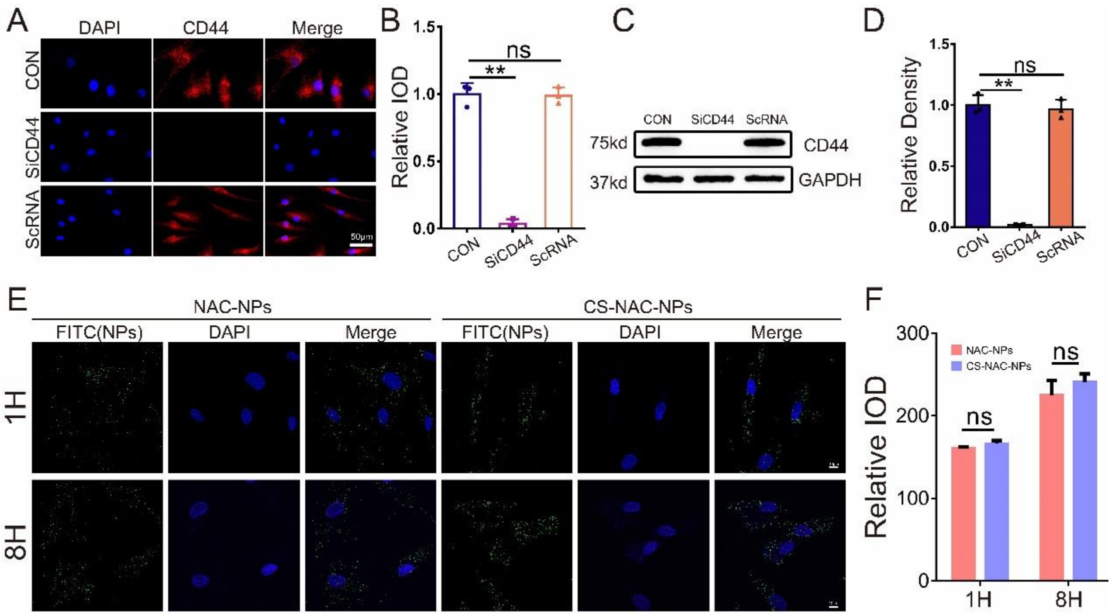

S Figure 1. CS-NAC-NPs specifically targeted chondrocytes through CD44 receptor–mediated interaction.

(A). Representative immunofluorescence images of CD44 in articular cartilage of the indicated groups. Scale bars, 50 μm. (B). Quantification of immunofluorescence analysis (n=3 for each group). (C). WB analysis of CD44. (D). Quantification of WB analysis (n=3 for each group). (E, F) Quantitative analysis of intracellular fluorescence intensity to assess nanoparticle uptake by chondrocytes transfected with SiCD4. Scale bar = 10 μm. Data represent mean ± SD; *P < 0.05, **P < 0.01. ns: not significant.

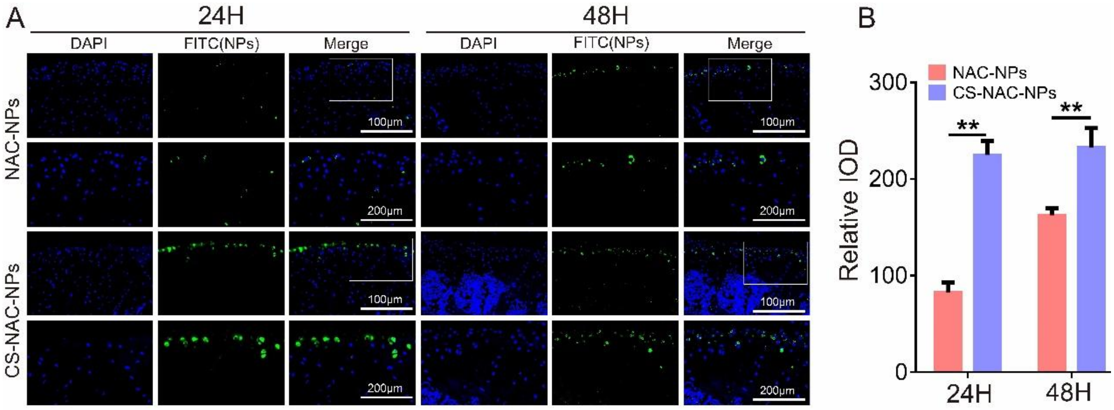

S Figure 2. CS-modified nanoparticles exhibited enhanced uptake by chondrocytes in vivo.

(A). Representative image of fluorescence of nanoparticles within the chondrocytes of the knee joint. Low Field, Scale bar = 100 μm. High Field, Scale bar =200 μm. (B). Quantitative analysis of intracellular fluorescence intensity to assess nanoparticle uptake by chondrocytes. Data represent mean ± SD; *$P < 0.05$, **$P < 0.01$.

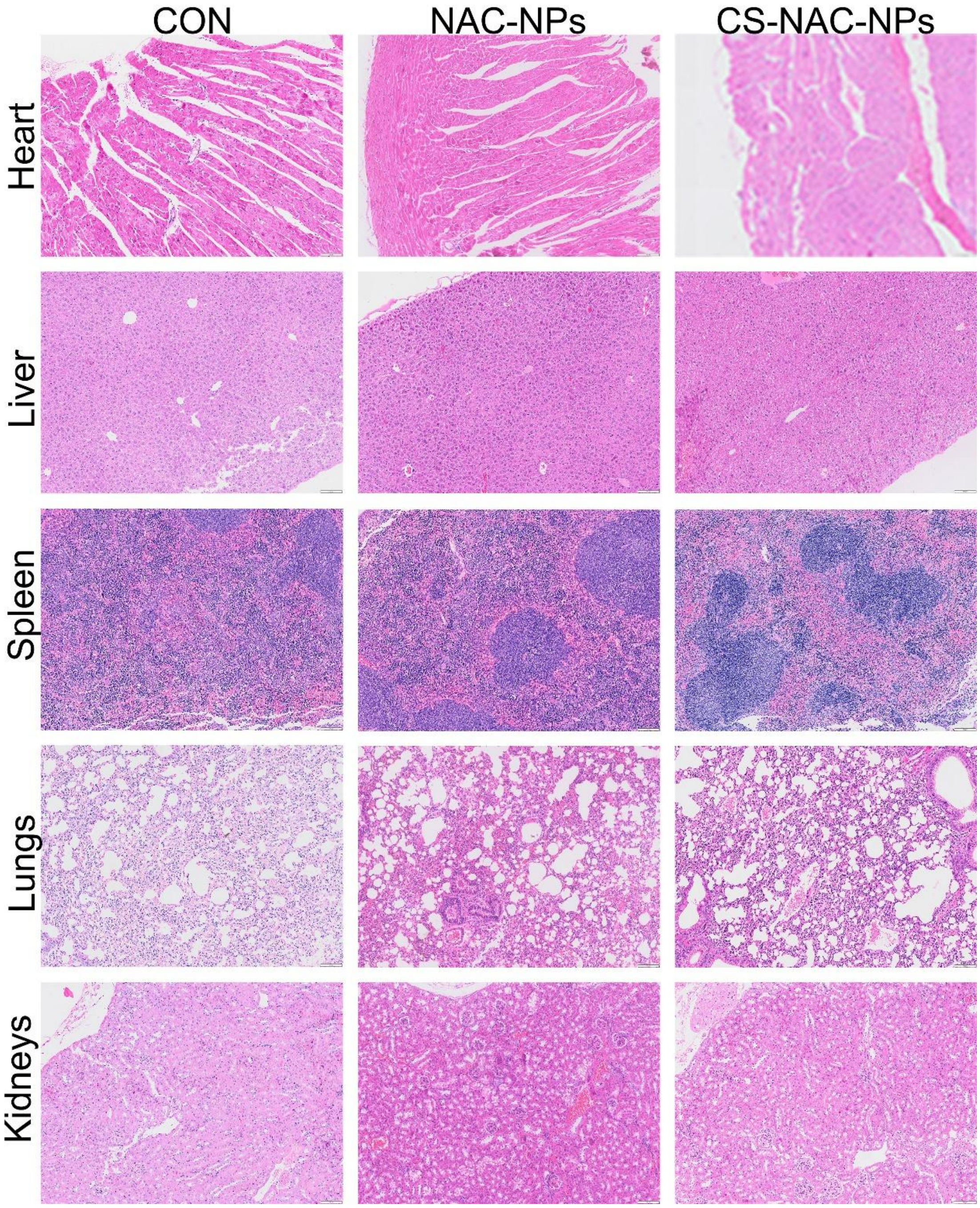

S Figure 3 Two types of nanoparticles have no internal organ toxicity

Thirty days after the injection of nanoparticles into the left knee joint (the CON group received the same amount of PBS), HE-stained sections of the heart, liver, spleen, lungs and kidneys were prepared. Scale bars, 100 μm.

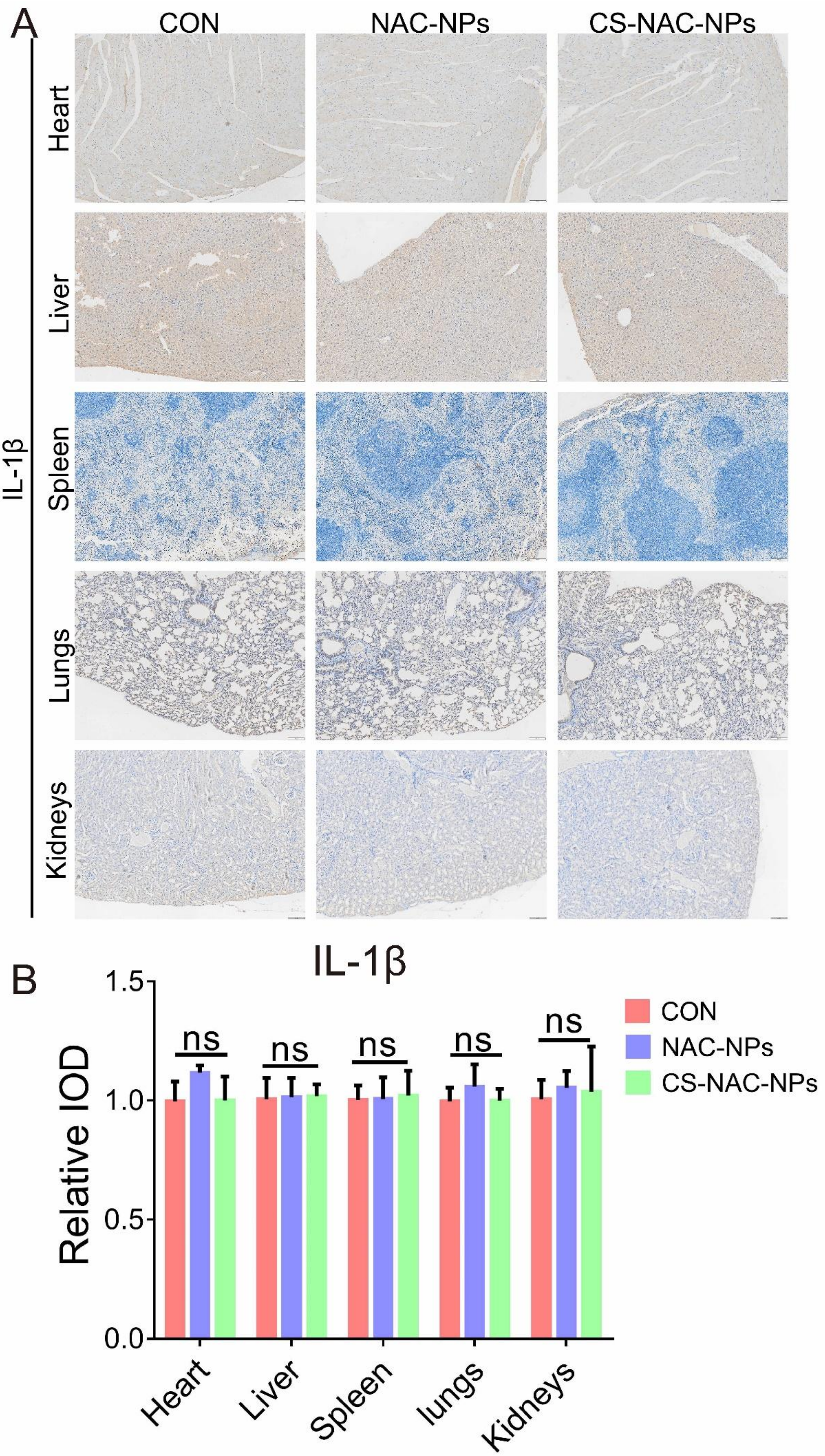
A
CON
NAC-NPs
CS-NAC-NPs
IL-1β
Heart
Liver
Spleen
Lungs
Kidneys
B
IL-1β
Relative IOD
1.5
1.0
0.5
0.0
ns
ns
ns
ns
ns
CON
NAC-NPs
CS-NAC-NPs
Heart
Liver
Spleen
lungs
Kidneys

S Figure 4 Two types of nanoparticles have no internal organ toxicity

(A). The representative immunohistochemical images of IL-1β in the heart, liver, spleen, lung and kidney sections of each group are shown above. Scale bars, 100 μm. (B). Quantification of immunohistochemical analysis (n=3 for each group). Data are presented as mean ± SD. ns: not significant.

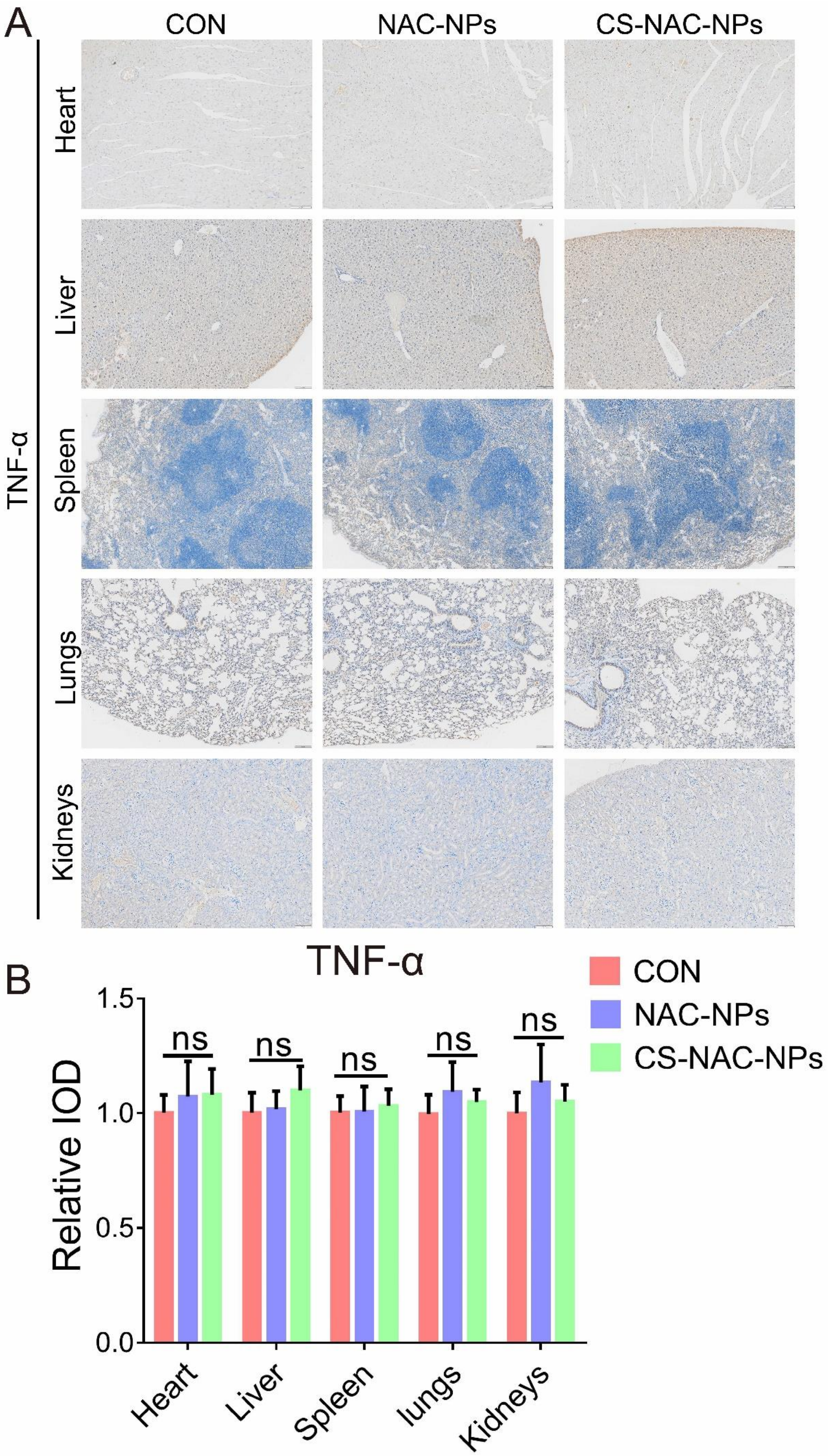
A
CON
NAC-NPs
CS-NAC-NPs
TNF-α
Heart
Liver
Spleen
Lungs
Kidneys
B
TNF-α
CON
NAC-NPs
CS-NAC-NPs
Relative IOD
1.5
1.0
0.5
0.0
ns
ns
ns
ns
ns
Heart
Liver
Spleen
lungs
Kidneys

S Figure 5 Two types of nanoparticles have no internal organ toxicity

(A). The representative immunohistochemical images of TNF-α in the heart, liver, spleen, lung and kidney sections of each group are shown above. Scale bars, 100 μm. (B). Quantification of immunohistochemical analysis (n=3 for each group). Data are presented as mean ± SD. ns: not significant.

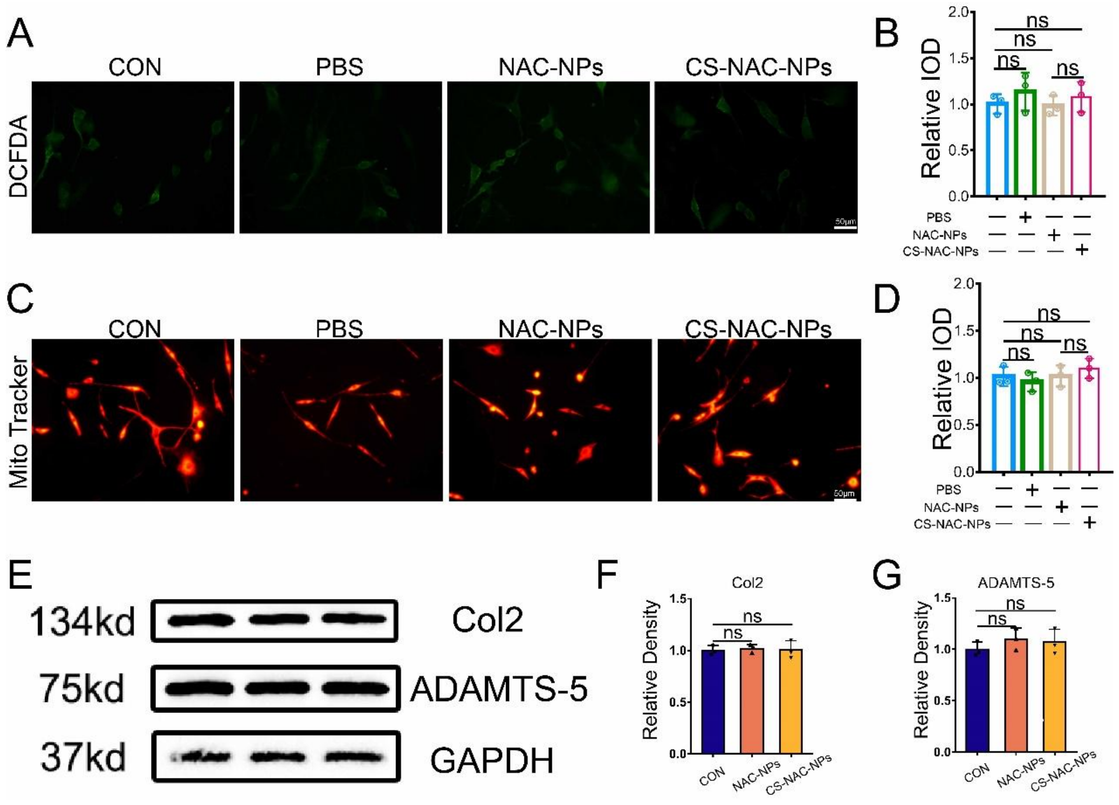

S Figure 6 The application of nanoparticles alone does not elicit any response from chondrocytes in terms of verification, metabolism, or oxidative stress.

(A) Representative images of ROS levels in chondrocytes. Scale bar = 50 μm. (B) Quantitative analysis of fluorescence intensity (n=3 for each group). (C) Representative fluorescence images of mitochondria in chondrocytes. Scale bar = 50 μm. (D) Quantitative analysis of fluorescence intensity (n=3 for each group). (E–G) Western blot analysis of Col2 and ADAMTS-5. Data represent mean ± SD; ns: not significant.

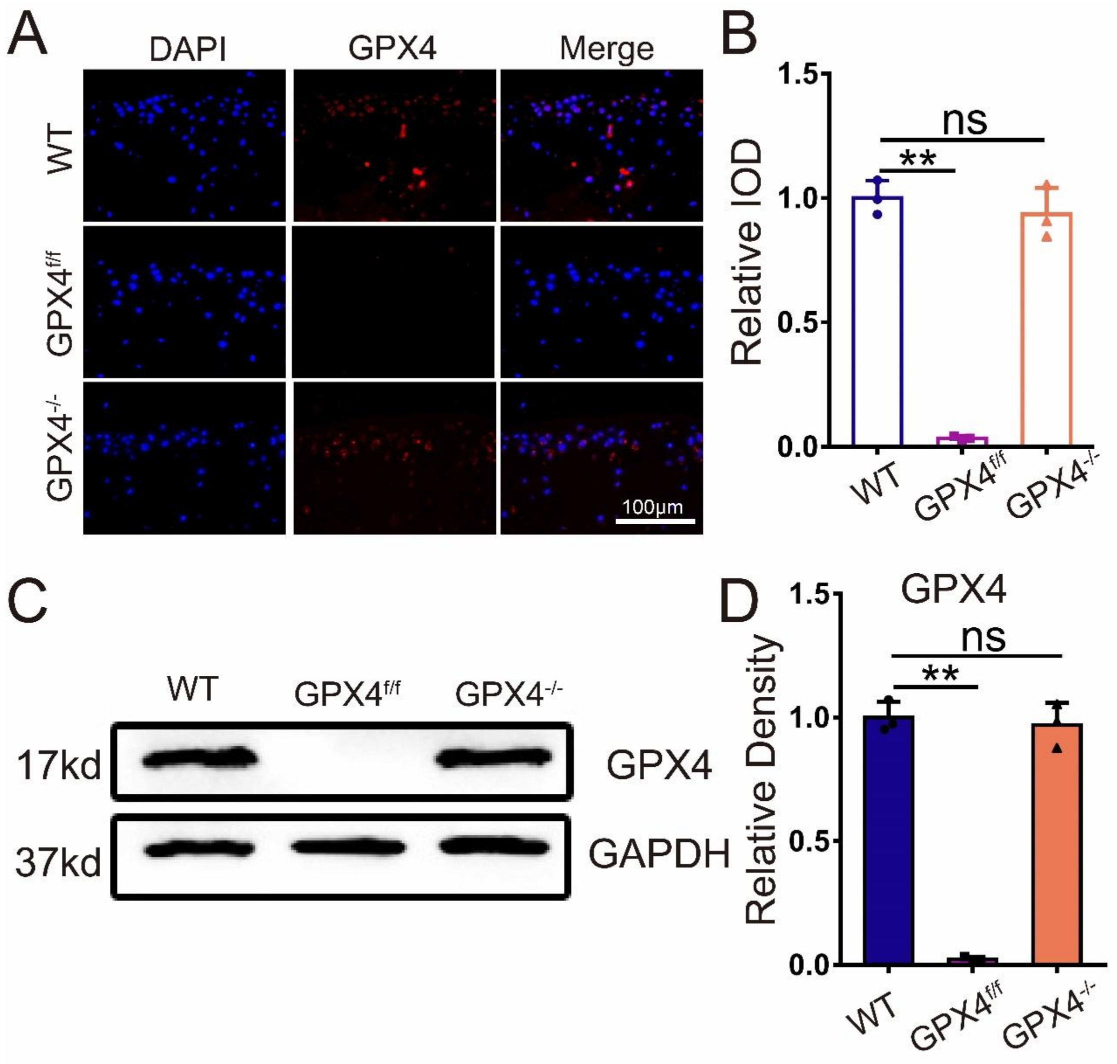

S Figure 7 Verification of GPX4 knockout efficiency in chondrocyte-specific tissues.

(A). Representative immunofluorescence images of GPX4 in articular cartilage of the indicated groups. Scale bars, 100 μm. (B). Quantification of immunofluorescence analysis (n=3 for each group). (C). WB analysis of GPX4. (D). Quantification of WB analysis (n=3 for each group). All data are presented as mean ± SD; *$p < 0.05$, **$p < 0.01$.

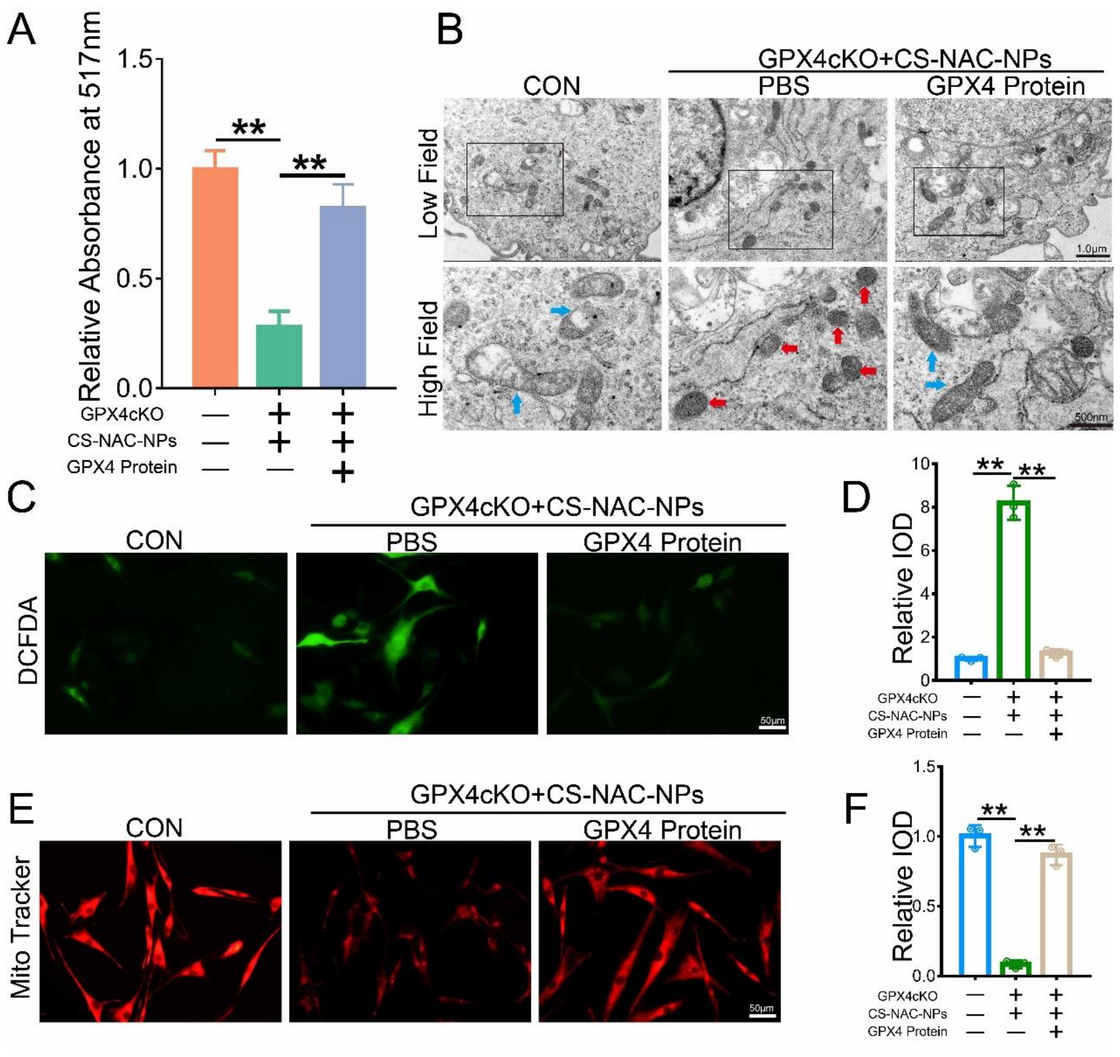

S Figure 8 CS-NAC-NPs inhibit ferroptosis in chondrocytes through the GPX4 protein and protect the activity of chondrocytes.

(A) Cell viability of GPX4 cartilage-specific knockout chondrocytes subjected to 1 MPa abnormal mechanical stress with or without CS-NAC-NPs or GPX4 Protein treatment for 24 h, assessed by a Calcein-AM/PI Viability/Cytotoxicity Assay Kit. (B) Transmission electron microscopy (TEM) images showing mitochondrial morphology; ferroptosis features such as condensed mitochondrial membranes and reduced cristae were observed. After the deletion of GPX4, the CS-NAC-NPs nanoparticles were unable to inhibit chondrocytes ferroptosis and the activity of chondrocytes decreased.

After supplementing with the GPX4 protein, the CS-NAC-NPs inhibited ferroptosis in chondrocytes and increased the activity of chondrocytes. Blue arrows show the normal mitochondria. Red arrows show the shrunken mitochondria. Scale bars, 1 μm (low field), 500 nm (high field). (C) Representative images of ROS levels in chondrocytes. Scale bar = 50 μm. (D) Quantitative analysis of fluorescence intensity (n=3 for each group). (E) Representative fluorescence images of mitochondria in chondrocytes. Scale bar = 50 μm. (F) Quantitative analysis of fluorescence intensity (n=3 for each group). All data are presented as mean ± SD; *$p < 0.05$, **$p < 0.01$.
